# ELTD1 inhibits differentiation of hemogenic endothelium progenitors from human embryonic stem cells through the HPIP–Wnt pathway

**DOI:** 10.1038/s12276-025-01473-6

**Published:** 2025-06-02

**Authors:** Qian Luo, Yijin Chen, Honghu Li, Yan Long, Wei Shan, Xiangjun Zeng, Shuyang Cai, Ye Meng, Cong Wei, Yulin Xu, Ruxiu Tie, Yi Luo, Pengxu Qian, Meng Zhang, He Huang

**Affiliations:** 1 Bone Marrow Transplantation Center of The First Affiliated Hospital & Liangzhu Laboratory, Zhejiang University School of Medicine, Hangzhou, China; 2 Institute of Hematology, Zhejiang University, Hangzhou, China; 3 Zhejiang Province Engineering Research Center for Stem Cell and Immunity Therapy, Hangzhou, China; 4 Department of Ultrasound in Medicine, The Second Affiliated Hospital of Zhejiang University School of Medicine, Zhejiang University, Hangzhou, China; 5 Research Center of Ultrasound in Medicine and Biomedical Engineering, The Second Affiliated Hospital of Zhejiang University School of Medicine, Zhejiang University, Hangzhou, China; 6 Shanghai Municipal Hospital of Traditional Chinese Medicine, Shanghai University of Traditional Chinese Medicine, Shanghai, China; 7 Department of Hematology, The Second Clinical Medical College, Shanxi Medical University, Taiyuan, China; 8 Department of Hematology, Taizhou Hospital of Zhejiang Province Affiliated to Wenzhou Medical University, Linhai, China

**Keywords:** Stem-cell differentiation, Embryonic stem cells

## Abstract

Human embryonic stem cells (hESCs) serve as an ideal cell source for generating hematopoietic stem cells (HSCs). In embryonic hematopoiesis, hemogenic endothelium has been identified as a source of HSCs, yet the regulatory mechanisms remain elusive. Here, through dynamic gene expression profiling analysis and verification, we find that ELTD1 expression parallels genes related to the specification of hemogenic endothelium progenitors (HEPs) from hESCs and is highly expressed in the HEPs. We then investigate the impact of ELTD1 on the hematopoietic differentiation of hESCs via gain- and loss-of-function experiments. Knockdown or deletion of ELTD1 mediates hESC hematopoiesis by specifically facilitating the generation of HEPs, thus promoting endothelial-to-hematopoietic transition to generate more hematopoietic cells. Besides, the overexpression of ELTD1 serves to further solidify this conclusion. Mechanistically, we demonstrate that ELTD1 exerts its function through the Wnt signaling pathway by bioinformatic analyses and functional studies. In addition, our results demonstrate a protein–protein interaction between ELTD1 and HPIP and further reveal that HPIP modulates the Wnt signaling pathway through LEF1. Collectively, these findings indicate that the ELTD1–HPIP–LEF1–Wnt regulatory axis acts as a novel mechanism regulating HEP generation during early hematopoietic differentiation of hESCs, providing new insights into the molecular mechanisms underlying human hematopoiesis.

## ELTD1 negatively regulates early hematopoiesis from hESCs

Hematopoietic stem cell (HSC) transplantation is used to treat blood cancers but faces challenges such as donor shortages and complications. Researchers are exploring human embryonic stem cells (hESCs) as an alternative source for these stem cells. In this study, researchers focused on a protein called ELTD1, which may play a role in forming hemogenic endothelium progenitors (HEPs), a key step in creating hematopoietic stem cells. They used genetic techniques to study ELTD1’s function in hESCs. By reducing ELTD1, they found an increase in HEPs and subsequent blood cell production. This suggests that ELTD1 negatively regulates early blood cell development by affecting the Wnt signaling pathway, a crucial communication system in cells. The study concludes that targeting ELTD1 could improve the generation of blood cells from hESCs, offering potential advancements in regenerative medicine.

This summary was initially drafted using artificial intelligence, then revised and fact-checked by the author.

## Introduction

Hematopoietic stem cell (HSC) transplantation has been applied in the treatment of hematological malignancies in the clinic. However, its application has been hindered by the shortage of donors and the occurrence of graft-versus-host disease after transplantation^1^. Therefore, there is an urgent need to find alternative sources of HSCs in vitro. Human embryonic stem cells (hESCs), which are generated from the inner cell mass of human embryos, possess multipotent differentiation potential and self-renewal capabilities^2^. Hematopoietic differentiation of hESCs has emerged as a promising strategy for generating HSCs. Despite recent advances in inducing hESC differentiation, the production of HSCs with engraftment potential from hESCs remains highly challenging. Therefore, unraveling the mechanisms governing hESC hematopoietic differentiation will offer insights into early human hematopoietic development and could also enhance the generation of HSCs from hESCs for clinical applications.

Hematopoietic differentiation of hESCs imitates the progression of hematopoietic development in vivo^3,4^. Generally, hematopoietic differentiation from hESCs involves a sequential series of cell fate decisions: the production of the mesoderm, the generation of specialized endothelial cells, termed hemogenic endothelium progenitors (HEPs), and finally, the emergence of hematopoietic cells from HEPs through the process of endothelial-to-hematopoietic transition (EHT)^5^. Each stage is under the regulation of extrinsic factors, intracellular signaling pathways and transcription factors. In addition, accumulating evidence demonstrates that G protein-coupled receptors (GPCRs), the largest family of membrane protein, play critical roles in hematopoietic differentiation. For instance, G protein-coupled receptor 183 (Gpr183) has been identified as a crucial factor for the emergence of hematopoietic stem and progenitor cells by suppressing Notch signaling before the initiation of EHT^6^. LGR4 enhances hESC hematopoiesis by facilitating mesoderm induction through the activation of TGF-β signaling^7^. Zebrafish embryos with suppressed Gpr56 expression exhibit substantial decreases in the generation of aortic hematopoietic stem and progenitor cells during EHT^8^. As mentioned earlier, hematopoietic cells originate from a specialized group of endothelial cells with hemogenic potential, known as the HEPs^9,10^. However, numerous GPCRs and their functions, as well as the associated mechanisms, have not been elucidated in HEP generation so far. Therefore, identification of novel GPCRs in directing hematopoietic differentiation, especially at the stage of HEPs, is essential for a better understanding of hematopoiesis and, consequently, the de novo generation of clinical-grade HSCs.

Recently identified as a novel adhesion GPCR (aGPCR) member, the epidermal growth factor latrophilin and seven transmembrane domain-containing protein 1 (ELTD1) has been found to be involved in brain angiogenesis^11,12^ and exhibits specific expression in endothelial cells across various tissues, as indicated by the Human Protein Atlas database. However, it remains unclear whether ELTD1 plays a role in human hematopoiesis, particularly in HEP generation. In this study, using genome-wide transcriptomic analysis combined with genetic gain-of-function and loss-of-function studies, we find that ELTD1 is essential for the HEP specification. We also found that ELTD1 modulates HEP formation through the HPIP–LEF1–Wnt axis. Our findings unveil the role of ELTD1 in hematopoietic differentiation, shedding light on previously undiscovered mechanisms governing human hematopoiesis. Moreover, our discoveries offer fresh insights into the generation of hematopoietic cells from hESCs, thereby presenting promising prospects for regenerative medicine.

## Materials and methods

### Cell culture and hematopoietic differentiation

The hESC line H1 utilized in this study was sourced from our laboratory’s repository. In addition, the hESC line H9 and induced pluripotent stem cells (iPSCs) were obtained from Ubigene Biosciences and UniXell Biotechnology, respectively. hESCs and iPSCs were cultured on Matrigel-coated (Corning) plates in mTeSR1 medium (Stem Cell Technologies). HEK293T cells were cultured in Dulbecco’s modified Eagle medium (Corning) supplemented with 10% fetal bovine serum (Gibco). Hematopoietic differentiation of hESCs or hiPSCs was performed using STEMdiff Hematopoietic Kit (Stem Cell Technologies), as previously described^13^. To be more specific, hESCs or iPSCs were passaged as aggregates with diameters ranging from 100 to 200 µm using ReLeSR (Stem Cell Technologies). On day 0, adherence of 4–10 colonies/cm^2^ was confirmed. The culture medium was aspirated and replaced with 1 ml of medium A per well, followed by incubation at 37 °C for 48 h. On day 2, 0.5 ml of the medium was gently removed, and 0.5 ml of fresh medium A was added, with subsequent incubation for an additional 24 h. On day 3, the medium was aspirated and replaced with 1 ml of medium B, and the cells were incubated for 48 h. Medium exchange was repeated on days 5 and 7 by carefully removing 0.5 ml of medium and adding 0.5 ml of fresh medium B, ensuring the preservation of floating cells, followed by incubation for 48 h. Cells can be collected at specific time points to accommodate the experimental requirements of different studies.

### Establishment of ELTD1-deleted hESC lines with iCRISPR–Cas9 system

The online CRISPR Design Tool (http://crispor.tefor.net/crispor.py) was used to design the single guide RNAs (sgRNAs) targeting ELTD1. Details of the inducible CRISPR (iCRISPR)–Cas9 system technology and ELTD1^−/−^ 1# clone information can be obtained from our previous report^14^. A second homozygous ELTD1 deletion clone (ELTD1^−/−^ 2#) was constructed using the same method, and its information and characteristics are shown in Supplementary Fig. 4f–h and Supplementary Fig. 5a–e.

### Alkaline phosphatase staining

The cells were treated with 4% paraformaldehyde for 10 min, rinsed with Dulbecco's phosphate-buffered saline (D-PBS) and incubated with 5-bromo-4-chloro-3-indolyl-phosphate (BCIP)–nitro blue chloride (NBT) for 30 min. After 30 min, BCIP–NBT was discarded and D-PBS was added to abort the reaction. The cells were observed under a light microscope (Nikon).

### Karyotype analysis

Karyotype analysis service was provided by VivaCell Biosciences.

### siRNA synthesis, plasmid construction and lentivirus production

The small interfering RNAs (siRNAs) of ELTD1, ELTD1-short hairpin RNA (shRNA) and ELTD1-overexpression (OE) plasmids were constructed and obtained from Transheep, and the efficiency of knockdown or overexpression was confirmed. The sequences used in this study are listed in Supplementary Table 1. siRNAs were transfected into cells using Lipofectamine 2000 according to the manufacturer’s protocols. ELTD1 knockdown or overexpressing hESCs were generated using the lentivirus produced in HEK293T cells. In brief, HEK293T cells of 60–80% confluent confluency were transfected with the psPAX2, pMD2.G, ELTD1-shRNA or ELTD1-OE plasmids using polyethylenimine. The supernatants were collected after 48 h and concentrated by ultracentrifugation at 4 °C, 25,000 rpm for 1.5 h. The lentivirus of HPIP-shRNA and LEF1-OE was obtained from Transheep. hESCs were infected with lentivirus at a multiplicity of infection of 10 for 6 h. After 6 h, the viral supernatants were discarded and the infected hESCs were cultured in fresh mTeSR1 medium (Stem Cell Technologies). Puromycin (1 μg/ml, Selleck) was applied to select for positive cells after 48 h.

### Flow cytometry and cell sorting

After dissociation with Accutase (Stem Cell Technologies), the cells were stained with the desired antibodies (Supplementary Table 2) for 20 min. Then, the cells were rinsed with D-PBS, filtered through a 70-μm cell strainer (Falcon) and analyzed by flow cytometry (DxFLEX, Beckman Coulter). Cell sorting was performed using a cell sorter (MoFlo Astrios EQ, Beckman Coulter). For apoptosis analysis, cells were stained with Annexin V and propidium iodide (PI) using an Annexin V-FITC apoptosis detection kit (BD Biosciences). Cells were dissociated into single-cell suspensions, washed twice with ice-cold PBS and resuspended in binding buffer at a concentration of 1 × 10^5^ cells per 100 µl. Then, 5 µl of Annexin V-FITC and 5 µl of PI were added to the cell suspension and incubated for 10 min at room temperature (RT) in the dark. Subsequently, 400 µl of 1× Binding Buffer was added to each sample, and flow cytometry analysis was performed within 1 h.

### Colony-forming unit (CFU) assay

A total of 1 × 10^4^ hematopoietic cells derived from iCas9-H1 and ELTD1^−/−^ hESCs were replated into MethoCult medium (Stem Cell Technologies). The cells were cultured for 12 days at 37 °C under 5% CO_2_ conditions to allow differentiation. Distinct hematopoietic lineage colonies were then counted and scored according to standard morphological criteria.

### Hematoxylin and eosin staining

Teratomas were fixed in 4% paraformaldehyde (Solarbio) at 25 °C, embedded in paraffin wax and sectioned into 3-μm slices. The sections were deparaffinized with three xylene treatments and rehydrated through a graded ethanol series. After a 2-min tap water rinse, the sections were stained with Gill’s hematoxylin V for 5 min and washed in tap water for 5 min and then in 95% ethanol for 2 min. Subsequently, they were stained with eosin Y for 1 min, dehydrated in ethanol and xylene and mounted with Canada balsam. Photomicrographs were obtained using a Pannoramic DESK (3DHISTECH).

### Immunofluorescence

The cells were fixed with 4% paraformaldehyde (Solarbio) for 20 min at RT, then permeabilized with a 0.1% Triton X-100 solution in PBS for an additional 20 min at RT. Subsequently, the cells were incubated with a blocking buffer containing 5% bovine serum albumin for 1 h at RT to prevent nonspecific binding of antibodies. After blocking, the cells were washed once with D-PBS, followed by incubation with the desired primary antibodies at 4 °C overnight. After primary antibody incubation, the cells were thoroughly washed three times with D-PBS for 5 min each. The cells were then incubated with the appropriate Alexa Fluor-conjugated secondary antibody (Thermo Fisher Scientific) for 1 h at RT in the dark to avoid photobleaching. Before observation, the cell nuclei were stained with 4′,6-diamidino-2-phenylindole (DAPI, Yeasen) for 10 min at RT, after which the cells were washed three times with D-PBS to remove excess DAPI. The cells were mounted with an anti-fade mounting medium to preserve fluorescence signals, and high-resolution images were captured using a confocal laser scanning microscope (LSM 880, Zeiss). Details regarding the antibodies used can be found in Supplementary Table 2.

### Western blotting

Cell lysates were subjected to gel electrophoresis on sodium dodecyl sulfate polyacrylamide gel electrophoresis (SDS–PAGE) and western blotting with the desired antibodies. The signals of the individual bands in the immunoblots were observed using an image analyzer (Clinx). The information of antibodies is presented in Supplementary Table 2.

### Real-time quantitative PCR (qPCR)

Total RNA was extracted using an RNA extraction kit (Vazyme) and transcribed into cDNA using a reverse transcription kit (Vazyme). All samples were tested with SYBR Green (Vazyme) in triplicate on a PCR cycler (CFX-96, Bio-Rad). The relative expression of individual genes was normalized to actin. The primers utilized in this study are detailed in Supplementary Table 1.

### Teratoma formation

Teratoma formation experiments were approved by the Laboratory Animal Center of Zhejiang University. All mice were bred and treated ethically under specific-pathogen‐free conditions. In brief, 1 × 10^6^ hESCs suspended in Matrigel diluted with D-PBS were injected into the gastrocnemius muscle of NOD-SCID mice. After 6–8 weeks, teratomas were collected and treated with 4% paraformaldehyde. Hematoxylin and eosin (H&E) staining was used to detect the three germ layer formation potentials of hESCs.

### RNA sequencing and bioinformatic analyses

RNA sequencing (RNA-seq) analysis was accomplished by BGI Company. Bulk RNA-seq data were converted to Transcripts Per Million (TPM) for gene expression analysis. Heatmaps were generated using R language. Gene set enrichment analysis (GSEA) and Kyoto Encyclopedia of Genes and Genomes (KEGG) pathway enrichment analyses were accomplished using the online tool of BGI.

### Co-immunoprecipitation, mass spectrometry and data processing

Cells were collected and lysed at 4 °C for 30 min with GPCR extraction and stabilization reagent (Thermo Fisher Scientific) supplemented with protease inhibition cocktail. Co-immunoprecipitation (Co-IP) was conducted by following manufacturers’ instructions with anti-Flag M2 magnetic beads (Sigma) at 4 °C overnight. After this, the beads were washed with precold Tris-Buffered Saline (TBS), and the pellets were resuspended in 2× loading buffer without reducing agents, then boiled at 100 °C for 3 min. The resulting supernatants were collected and analyzed using western blotting. Proteins that were immunoprecipitated from cell lines expressing Flag-ELTD1 were isolated using anti-Flag M2 magnetic beads, separated by SDS–PAGE and then subjected to silver staining (Beyotime). After this, the gel was excised from the samples and subjected to tryptic digestion according to a standard protocol^15^. The tryptic peptides were dissolved and the separated peptides were analyzed using Orbitrap Exploris 480 with a nano-electrospray ion source (Thermo Fisher Scientific). Next, PD search engine (v.2.4) software was used to analyze the mass spectrometry (MS) data.

### Molecular docking analysis

Rigid protein–protein docking was performed to investigate the relationship between ELTD1 and HPIP by using GRAMM-X (http://gramm.compbio.ku.edu/). The protein structural domains of ELTD1 and HPIP were obtained from the Protein Data Bank PDB database (http://www.rcsb.org/). Pymol (Version 2.4) and PDBePISA (https://www.ebi.ac.uk/pdbe/pisa/) were used to examine protein–protein interactions and further visual analysis.

### Statistical analysis

Statistical analyses were performed using SPSS software. Statistical differences were examined using Student’s *t-*test or one-way analysis of variance. *P* < 0.05 was considered statistically significant.

## Results

### ELTD1 is a potential regulator of HEPs during early hematopoietic differentiation of hESCs

Hematopoietic differentiation of hES cells mimics human hematopoiesis in vivo and progresses through three sequential stages: mesoderm induction, HEP derivation and generation of hematopoietic cells^16^. These stages are distinguished by the expression of specific markers: CD309^17^ and APLNR^18^ for mesoderm induction, CD31 and CD34^7,19,20^ for HEP derivation, and CD43^21^ for hematopoietic cell generation. Therefore, we induced directed hematopoietic differentiation of hESCs using a previously reported strategy^13^ and assessed the temporal expression of these markers through qPCR, which showed dynamic changes during hESC differentiation. In addition, we analyzed the expression of markers associated with axial mesoderm (SFRP2 and DCLK1), paraxial mesoderm (MESP2 and MSGN1), intermediate mesoderm (CDH5 and OSR1), lateral plate mesoderm (CD309 and HAPLN1) and extraembryonic mesoderm (IGF2 and LUM). Notably, with the exception of the lateral plate mesoderm marker, the expression levels of all other mesoderm subtype markers were either suppressed or remained inactive on day 3 of differentiation. Moreover, the expression of pluripotent markers (OCT4 and SOX2) exhibited a gradual decline as differentiation progressed (Supplementary Fig. 1a). These results validate the effectiveness of our hematopoietic differentiation method in this study. In addition, we tested the alkaline phosphatase activity in cells of different days, which further confirmed the differentiation of hESCs (Supplementary Fig. 1b).

**Fig. 1:**
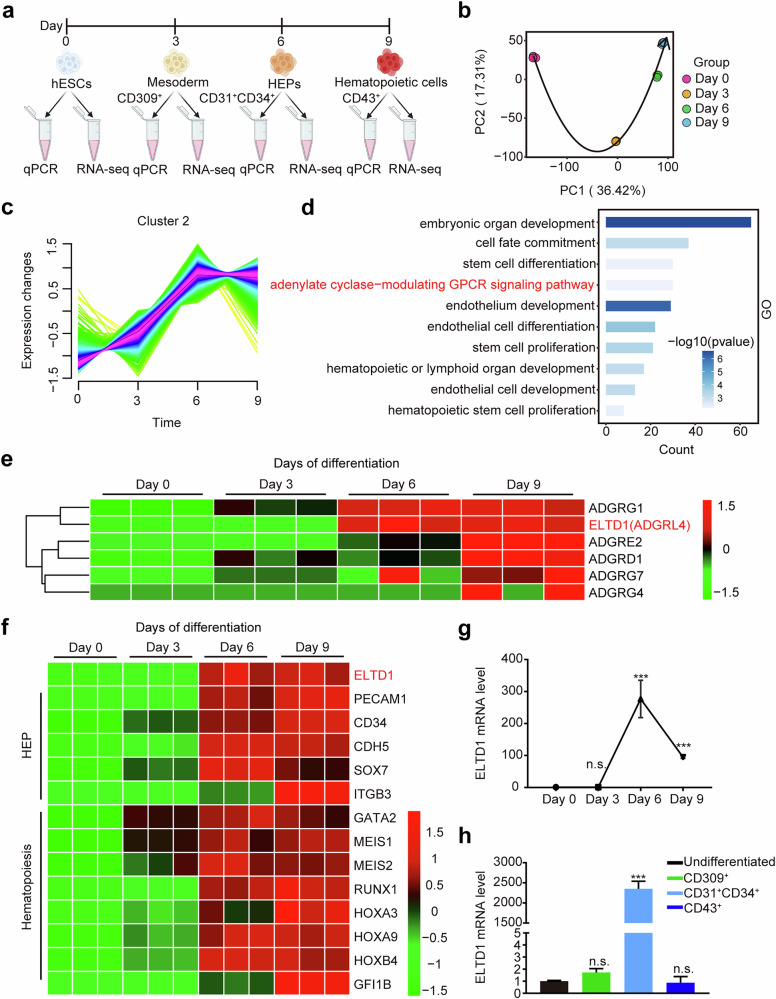
ELTD1 is a potential regulator of HEPs during early hematopoietic differentiation of hESCs. **a** A schematic overview of early hematopoietic differentiation process from hESCs and our experimental workflow. The process typically begins with the formation of mesoderm, followed by the emergence of HEPs, which eventually give rise to hematopoietic cells. **b** Principal component analysis (PCA) was applied to the RNA-seq dataset to visualize the overall similarities and differences in gene expression profiles across various time points. **c** Mfuzz analysis identified the patterns of gene expression in the time-course datasets of cluster 2. **d** GO analysis was performed to identify the significantly enriched biological processes in cluster 2. **e** A heatmap of dynamic aGPCR expression levels in the enriched GPCR signaling pathway during early hematopoietic differentiation from hESCs. **f** A heatmap showing dynamic gene expression associated with HEPs (for example, PECAM1 and CD34) and hematopoiesis (for example, GATA2 and RUNX1) during early hematopoietic differentiation from hESCs. **g** Time course analysis of ELTD1 mRNA level with qPCR during hESC hematopoietic differentiation. Results are shown as mean ± s.d.; *n* = 3. n.s., not significant, *P < 0.05, **P < 0.01, ***P < 0.001, compared with the day 0 group. **h** qPCR analysis of ELTD1 mRNA levels in undifferentiated hESCs, sorted CD309^+^ mesoderm, sorted CD31^+^CD34^+^ HEPs and sorted CD43^+^ hematopoietic cells derived from hESCs. Results are shown as mean ± s.d.; *n* = 3. n.s., not significant, *P < 0.05, **P < 0.01, ***P < 0.001, compared with the undifferentiated group.

To discover novel regulators of early hematopoietic differentiation of hESCs, we collected RNA samples at days 0, 3, 6 and 9 during the differentiation process for time-course RNA-seq analysis (Fig. 1a). Our transcriptome data showed high reproducibility among different biological replicates and reflected the continuity of hematopoietic differentiation process (Fig. 1b). To define the temporal characteristics of the transcript dataset during hESC hematopoietic differentiation, transcripts were grouped into six clusters by Mfuzz based on their expression levels (Fig. 1c and Supplementary Fig. 2). In these clusters, cluster 1 and 5 represented downregulated transcripts, while cluster 2, 3 and 6 represented upregulated transcripts. Transcripts in cluster 4 exhibited an increase from day 0 to day 3, subsequently decreasing significantly from day 3 to day 9. In short, the Mfuzz analysis showed notable differences in the relative abundance of transcripts across different days.

In the current study, we aim to identify potential regulators of HEP during hESC hematopoietic differentiation. Therefore, we focused on cluster 2 because the transcripts in this cluster showed a sharp upregulation from day 3 to day 6, corresponding to the stage of HEP generation (Fig. 1c). We then conducted a Gene Ontology (GO) analysis of cluster 2 and found that most of the transcripts in cluster 2 were associated with cell fate commitment, stem cell differentiation, endothelium development, GPCR signaling pathway, hematopoietic or lymphoid organ development, suggesting the reliability of candidate selection strategy (Fig. 1d). Among these, the GPCR signaling pathway was especially interesting because aGPCRs, the second-largest family of GPCRs, are broadly distributed and are crucial in regulating numerous developmental processes, including the orderly progression of hematopoiesis^22^. Approximately 30% of human aGPCRs are expressed in hematopoietic stem, progenitor or mature cells, where they define different cell populations^23^. Therefore, we analyzed aGPCRs of the enriched GPCR signaling pathway and found that ADGRG1 and ELTD1 were highly expressed at day 6, namely the HEP stage (Fig. 1e). As the indispensable effect of ADGRG1 in hematopoietic development has been previously reported^24^, we selected ELTD1 for further study.

ELTD1, also known as ADGRL4, is specifically expressed in endothelial cells of various tissues, as indicated by The Human Protein Atlas database (Supplementary Fig. 3a,b). We analyzed the dynamic changes of ELTD1 expression during hematopoietic differentiation and found that it differed from the other three members of the ADGRL family (Supplementary Fig. 3c). However, its expression pattern was parallel to the genes related to HEPs (ITGB3^20^, PECAM1^25^, CD34^26^ and SOX7^27^), as well as genes associated with hematopoiesis (MEIS1^16^, GATA2^28^, MEIS2^29^, RUNX1^30^, HOXA3^31^ and HOXA9^32^) (Fig. 1f). In accordance with the RNA-seq results, ELTD1 is highly expressed at day 6 of differentiation, with a slight decrease thereafter, as confirmed by qPCR analysis (Fig. 1g). To further confirm the results, we sorted CD309^+^ mesoderm cells, CD31^+^CD34^+^ HEPs and CD43^+^ hematopoietic cells at days 3, 6 and 9 and performed qPCR (Fig. 1a). As shown in Fig. 1h, ELTD1 expression was low in hESCs and mesoderm cells, peaked in the HEPs and then began to downregulate in hematopoietic cells. Collectively, these results suggest that ELTD1 may act as a regulator of HEPs during early hematopoietic differentiation of hESCs.

### ELTD1 suppression enhances hematopoietic differentiation of hESCs

**Fig. 2:**
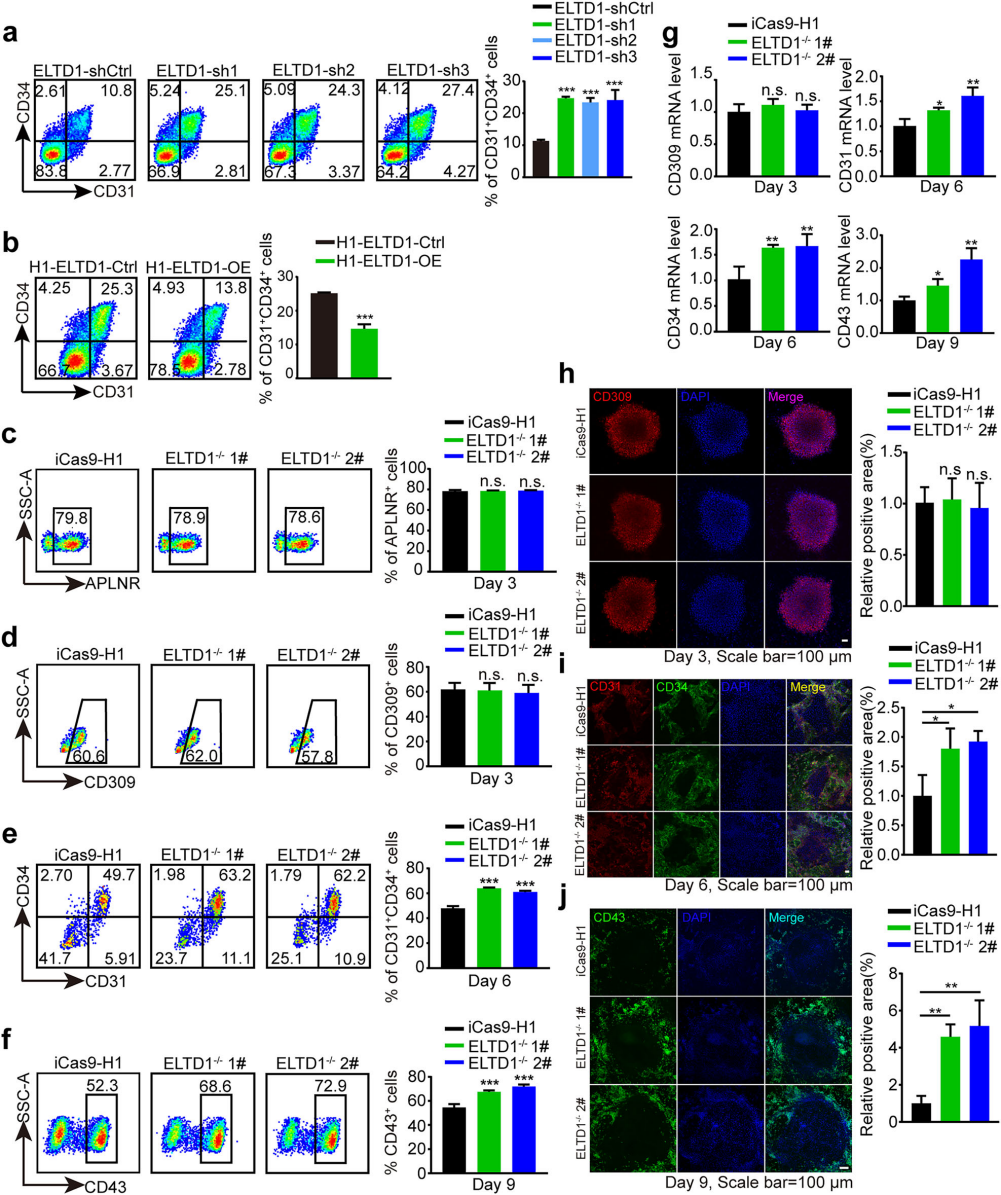
ELTD1 suppression enhances hematopoietic differentiation of hESCs. **a** Flow cytometry analysis of HEPs at day 6 of early hematopoietic differentiation. The percentage of CD31^+^CD34^+^ cells was quantified to assess the efficiency of HEP formation. Results are shown as mean ± s.d.; *n* = 3. n.s., not significant, *P < 0.05, **P < 0.01, ***P < 0.001, compared with the ELTD1-shControl (shCtrl) group. **b** Flow cytometry analysis of HEPs at day 6 of early hematopoietic differentiation. The percentage of CD31^+^CD34^+^ cells was quantified to assess the efficiency of HEP formation. Results are shown as mean ± s.d.; *n* = 3. n.s., not significant, *P < 0.05, **P < 0.01, ***P < 0.001, compared with the H1-ELTD1-Ctrl group. **c** Flow cytometry analysis of APLNR^+^ mesoderm cells at day 3 of early hematopoietic differentiation. Results are shown as mean ± s.d.; *n* = 3. n.s., not significant, *P < 0.05, **P < 0.01, ***P < 0.001, compared with the iCas9-H1 group. **d** Flow cytometry analysis of CD309^+^ mesoderm cells at day 3 of early hematopoietic differentiation. Results are shown as mean ± s.d.; *n* = 3. n.s., not significant, *P < 0.05, **P < 0.01, ***P < 0.001, compared with the iCas9-H1 group. **e** Flow cytometry analysis of HEPs at day 6 of early hematopoietic differentiation. The percentage of CD31^+^CD34^+^ cells was quantified to assess the efficiency of HEP formation. Results are shown as mean ± s.d.; *n* = 3. n.s., not significant, *P < 0.05, **P < 0.01, ***P < 0.001, compared with the iCas9-H1 group. **f** Flow cytometry analysis of hematopoietic cells at day 9 of early hematopoietic differentiation. The percentage of CD43^+^ cells was quantified to assess the proportion of hematopoietic cells. Results are shown as mean ± s.d.; *n* = 3. n.s., not significant, *P < 0.05, **P < 0.01, ***P < 0.001, compared with the iCas9-H1 group. **g** qPCR analysis of CD309 and APLNR at day 3, CD31 and CD34 at day 6, and CD43 at day 9 of early hematopoietic differentiation. Results are shown as mean ± s.d.; *n* = 3. n.s., not significant, *P < 0.05, **P < 0.01, ***P < 0.001, compared with the iCas9-H1 group. **h** Representative immunostaining of CD309^+^ (red) mesoderm cells and quantification of positive area (%) at day 3 of early hematopoietic differentiation. Nuclei were counterstained with DAPI (blue). Scale bar, 100 μm. Results are shown as mean ± s.d.; *n* = 3. n.s., not significant, *P < 0.05, **P < 0.01, ***P < 0.001, compared with the iCas9-H1 group. **i** Representative immunostaining of CD31^+^ (red) and CD34^+^ (green) HEPs and quantification of positive area (%) at day 6 of early hematopoietic differentiation. Nuclei were counterstained with DAPI (blue). Scale bar, 100 μm. Results are shown as mean ± s.d.; *n* = 3. n.s., not significant, *P < 0.05, **P < 0.01, ***P < 0.001, compared with the iCas9-H1 group. **j** Representative immunostaining of CD43^+^ (green) hematopoietic cells and quantification of positive area (%) at day 9 of early hematopoietic differentiation. Nuclei were counterstained with DAPI (blue). Scale bar, 100 μm. Results are shown as mean ± s.d.; *n* = 3. n.s., not significant, *P < 0.05, **P < 0.01, ***P < 0.001, compared with the iCas9-H1 group.

To investigate the potential role of ELTD1 in HEP specification, we generated ELTD1-knockdown hESs (Supplementary Fig. 4a). We used the ELTD1-knockdown hESCs to undergo hematopoietic differentiation. Flow cytometric analysis showed that transgenic shELTD1-hESCs displayed increased production of CD31^+^CD34^+^ HEPs at day 6 compared with control hESCs (Fig. 2a). To further confirm the results, transgenic ELTD1-expressing hESC lines were generated using lentivirus infection. Stable ectopic expression of ELTD1 and pluripotency analysis were confirmed thereafter (Supplementary Fig. 4b–d). As expected, ELTD1 overexpression significantly inhibited the generation of CD31^+^CD34^+^ HEPs at day 6 (Fig. 2b), resulting in outcomes opposite to those observed with ELTD1 knockdown. These findings suggest that ELTD1 may play a key role in regulating HEP generation from hESCs.

To gain a deeper understanding of ELTD1 in hematopoietic differentiation of hESCs, we disrupted ELTD1 in hESCs using the iCRISPR–Cas9 system, in which high expression of Cas9 protein can be induced by doxycycline treatment^33^. sgRNAs targeting exon 2 of ELTD1 were designed (Supplementary Fig. 4e), transfected and evaluated for genome editing efficacy using the T7E1 assay. By taking advantage of this system, two hESC homozygous ELTD1-deleted clones were constructed and named ELTD1^−/−^ 1# and ELTD1^−/−^ 2#, respectively. The deleted region of the sgRNA targeting site was confirmed using Sanger sequencing and PCR analysis. The results of ELTD1^−/−^ 1# has been documented in our previous report^14^, and the results of ELTD1^−/−^ 2# are shown in Supplementary Fig. 4f–h.

ELTD1^−/−^ 1# and ELTD1^−/−^ 2# exhibited compact and normal hESC morphologies (Supplementary Fig. 5a), and their pluripotency was retained by the high expression of OCT4, SOX2 and NANOG, which was assessed by qPCR (Supplementary Fig. 5b) and western blotting (Supplementary Fig. 5c). Moreover, ELTD1^−/−^ 1# and ELTD1^−/−^ 2# were injected into immunodeficient mice to form teratomas, verifying the multilineage differentiation potential after ELTD1 deletion (Supplementary Fig. 5d). Karyotype analysis was additionally performed on both cell lines. The results revealed a normal karyotype with no detectable chromosomal abnormalities in the analyzed cells (46, XY). We have previously documented the results of ELTD1^−/−^ 1# in our report^14^, and the results for ELTD1^−/−^ 2# are depicted in Supplementary Fig. 5e. These findings indicate that the gene knockout did not induce notable chromosomal alterations. In summary, ELTD1^−/−^ 1# and ELTD1^−/−^ 2# were considered suitable for subsequent studies on hESC hematopoietic differentiation.

To dissect the effect of ELTD1 deletion on hematopoiesis, we induced the iCas9-H1, ELTD1^−/−^ 1# and ELTD1^−/−^ 2# hESCs to go through hematopoietic differentiation and measured the generation of stage-specific cell populations. The expression of pluripotency genes was assessed and found to gradually decrease during hematopoietic differentiation, indicating a steady progression of differentiation (Supplementary Fig. 5f). Interestingly, no distinct discrepancy in the percentage of CD309^+^ and APLNR^+^ mesoderm cells was observed with ELTD1 ablation at day 3 (Fig. 2c, d). However, the generation of CD31^+^CD34^+^ HEPs at day 6, arising from mesoderm cells, was profoundly elevated by ELTD1 deletion (Fig. 2e), consistent with our prior observations in ELTD1-knockdown hESCs (Fig. 2a). As expected, flow cytometry analysis also showed an increase in CD43^+^ hematopoietic cells generated from HEPs (Fig. 2f). Moreover, the above results have also been confirmed by qPCR (Fig. 2g) and immunofluorescence analysis (Fig. 2h–j). To further validate this phenotype, we conducted knockdown and overexpression of ELTD1 in an additional hESC line H9 and in iPSCs. Subsequent hematopoietic differentiation experiments using the established stable cell lines consistently recapitulated the phenotypes observed previously, thereby further substantiating our findings (Supplementary Figs. 6 and 7). Together, these findings suggest that ELTD1 acts as a regulator of hematopoietic differentiation of hESCs, and its suppression could facilitate hematopoiesis.

### Inhibition of ELTD1 promotes hematopoietic differentiation of hESCs, particularly HEP specification

**Fig. 3:**
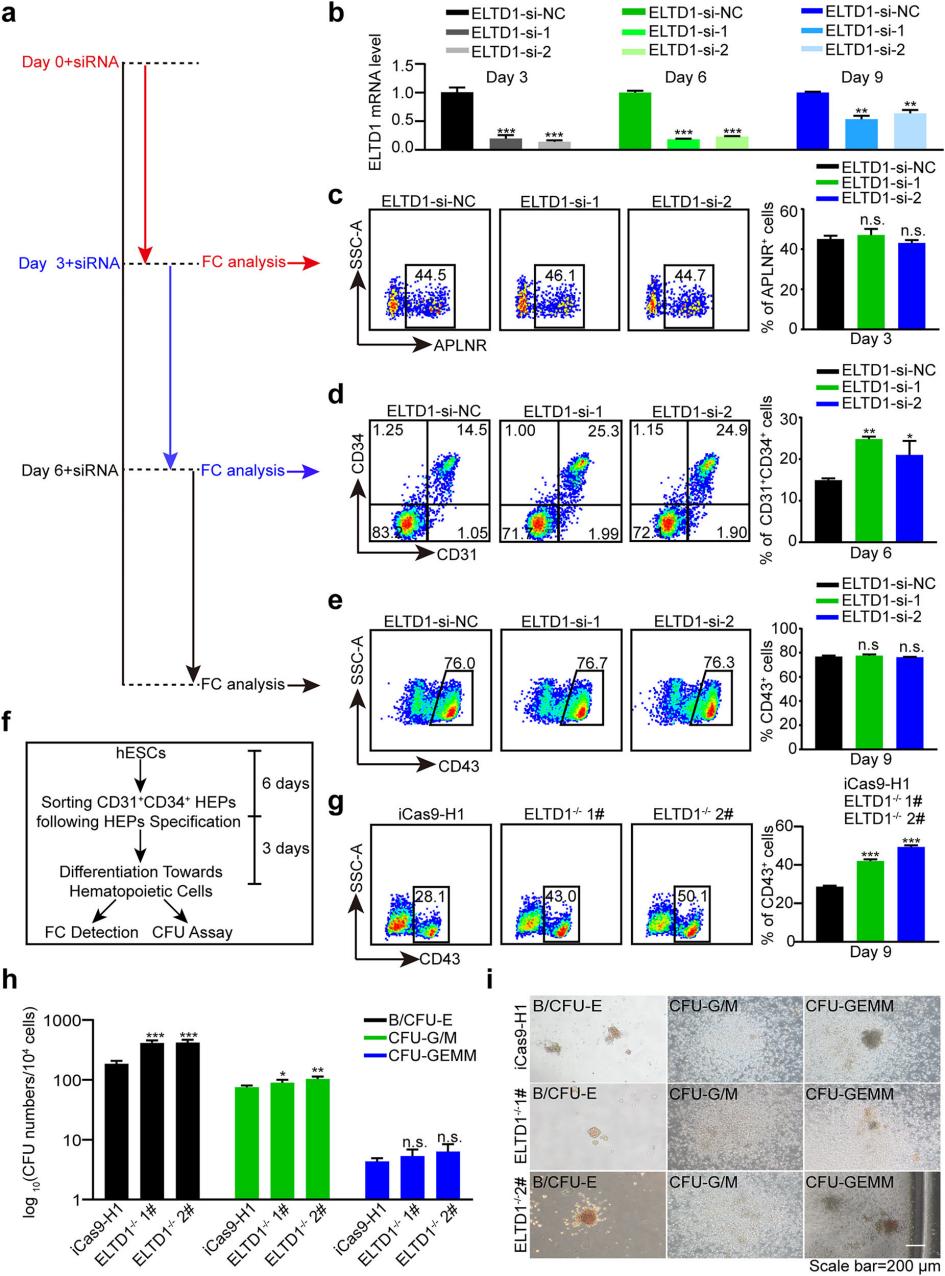
Inhibition of ELTD1 promotes hematopoietic differentiation of hESCs, particularly HEP specification. **a** A schematic diagram of the experimental timeline and workflow. siRNAs targeting ELTD1 or nontargeting control (si-NC) were transfected into cells on day 0, day 3 and day 6. Flow cytometry analysis was performed on day 3, day 6 and day 9 to assess the effects of ELTD1 knockdown on the generation of mesoderm, HEPs and hematopoietic cells, respectively. **b** qPCR analysis of ELTD1 transcript knockdown efficiency in differentiated cells. The mRNA levels of ELTD1 were measured to confirm the efficiency of siRNA-mediated knockdown. Results are shown as mean ± s.d.; *n* = 3. n.s., not significant, *P < 0.05, **P < 0.01, ***P < 0.001, compared with the si-NC group. **c** Flow cytometry analysis of APLNR^+^ mesoderm cells at day 3 after siRNA transfection. Cells were transfected with siRNAs on day 0, and the percentage of APLNR^+^ mesoderm cells was quantified by flow cytometry on day 3. Results are shown as mean ± s.d.; *n* = 3. n.s., not significant, *P < 0.05, **P < 0.01, ***P < 0.001, compared with the ELTD1-si-NC group. **d** Flow cytometry analysis of CD31^+^CD34^+^ HEPs at day 6 after siRNA transfection. Cells were transfected with siRNAs on day 3, and the percentage of CD31^+^CD34^+^ HEPs was quantified by flow cytometry on day 6. Results are shown as mean ± s.d.; *n* = 3. n.s., not significant, *P < 0.05, **P < 0.01, ***P < 0.001, compared with the ELTD1-si-NC group. **e** Flow cytometry analysis of CD43^+^ hematopoietic cells at day 9 after siRNA transfection. Cells were transfected with siRNAs on day 6, and the percentage of CD43^+^ hematopoietic cells was quantified by flow cytometry on day 9. Results are shown as mean ± s.d.; *n* = 3. n.s., not significant, *P < 0.05, **P < 0.01, ***P < 0.001, compared with the ELTD1-si-NC group. **f** A schematic diagram of the experimental timeline and workflow. CD31^+^CD34^+^ HEPs were sorted at day 6 of differentiation and assessed for their capacity to differentiate into hematopoietic cells, as well as subsequent multilineage differentiation potential. **g** Flow cytometry analysis of CD43^+^ hematopoietic cells at day 9. These cells were derived from sorted CD31^+^CD34^+^ HEPs at day 6, after 3 days of hematopoietic differentiation. Results are shown as mean ± s.d.; *n* = 3. n.s., not significant, *P < 0.05, **P < 0.01, ***P < 0.001, compared with the iCas9-H1 group. **h** The distribution of different colony types generated from iCas9-H1 or ELTD1^−/−^ hESCs. B/CFU-E, CFU-G/M and CFU-GEMM were documented and calculated. Results are shown as mean ± s.d.; *n* = 3. n.s., not significant, *P < 0.05, **P < 0.01, ***P < 0.001, compared with the iCas9-H1 group. **i** Representative images of different colony types from CFU assay. Day 9 floating cells were seeded in MethoCult medium for 12 days. Scale bar, 200 μm.

To explore at which stage ELTD1 functions in hematopoietic differentiation of hESCs, we transfected siRNAs at day 0, 3 and 6 of hematopoietic differentiation, respectively (Fig. 3a). As shown in Fig. 3b, ELTD1 was effectively suppressed by ELTD1-siRNAs. We assessed the changes in specific cell populations at days 3, 6 and 9. The results revealed no changes in the generation of mesoderm cells at day 3 and hematopoietic cells at day 9 (Fig. 3c, e). However, there was an enhancement in HEP production at day 6 (Fig. 3d). To further investigate the effects of ELTD1 on HEPs, we examined the apoptosis levels of CD31^+^CD34^+^ HEPs on day 6 of hematopoietic differentiation in both knockout and overexpression models. The results showed that neither the knockout nor the overexpression of ELTD1 affected the apoptosis levels of CD31^+^CD34^+^ HEPs (Supplementary Fig. 8a). Next, we sorted CD31^+^CD34^+^ HEPs on day 6 of differentiation and assessed their capacity to differentiate into hematopoietic cells (Fig. 3f). Compared with the iCas9-H1 group, the sorted CD31^+^CD34^+^ HEPs from the knockout group exhibited a significantly increased capacity to generate hematopoietic cells (Fig. 3g and Supplementary Fig. 8b). Moreover, we performed CFU assays to assess the differentiation potential in each group. The results showed that the knockout group was able to completely differentiate into B/CFU-E (burst-forming unit-erythroid/CFU-erythroid), CFU-G/M (CFU-granulocyte/macrophage) and CFU-GEMM (CFU-granulocyte/erythroid/macrophage/monocyte), similar to the iCas9-H1 group (Fig. 3h, i). Overall, these findings indicate that suppression of ELTD1 enhances hematopoietic differentiation of hESCs by promoting HEP production, thereby improving the subsequent generation of hematopoietic cells and their differentiation into downstream blood cell lineages.

### ELTD1 deletion suppresses the Wnt signaling during HEP generation

**Fig. 4:**
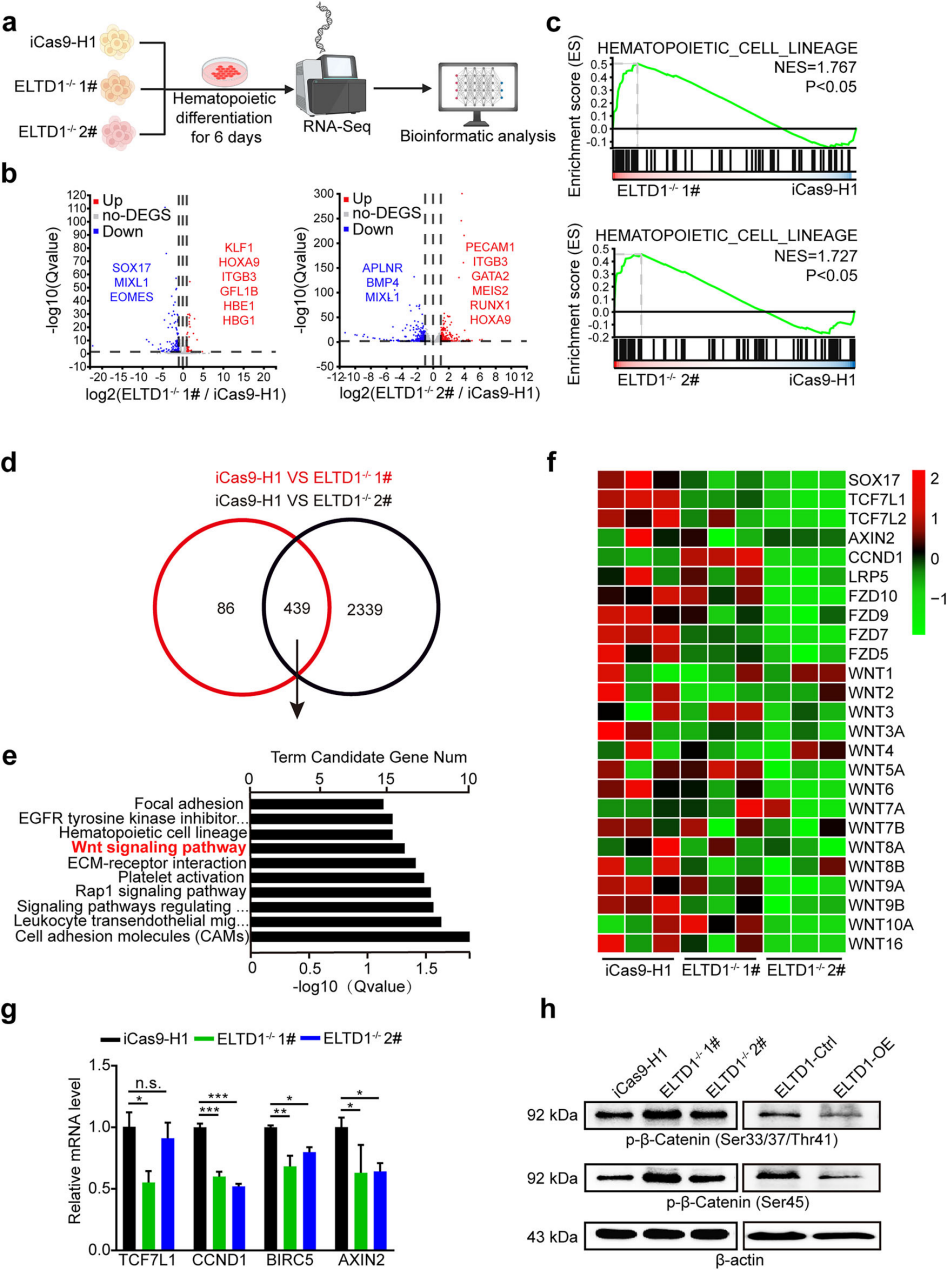
ELTD1 deletion suppresses the Wnt signaling during HEP generation. **a** A schematic diagram of the experimental workflow. **b** A volcano plot displaying the DEGs between ELTD1-deleted and control groups. Genes with significant upregulation (red) or downregulation (blue) are displayed. **c** GSEA reveals the enrichment of gene sets associated with ‘hematopoietic cell lineages’ in ELTD1-deleted cells compared with controls. The normalized enrichment score (NES) and *P* value are indicated. **d** The strategy of screening potential genes for further analysis. **e** A bar plot showing the top enriched KEGG pathways enrichment analysis of the screened candidate genes. The significance of enrichment is indicated by the −log_10_(*Q* value). **f** A heatmap showing the expression levels of Wnt signaling-related genes in ELTD1-deleted and control groups at day 6 of differentiation. **g** The mRNA levels of key Wnt signaling-related genes quantified by qPCR. Results are shown as mean ± s.d. *n* = 3. n.s., not significant, *P < 0.05, **P < 0.01, ***P < 0.001. **h** Protein levels of phosphorylated β-catenin (at serine 33, 37 and 45; tyrosine 41), an indicator of β-catenin degradation, were assessed by western blotting in ELTD1-KO or ELTD1-OE groups at day 6 of hematopoietic differentiation.

To dissect the molecular mechanism underlying ELTD1 regulation of HEPs, the differentiated cells at day 6 were collected for RNA-seq analysis (Fig. 4a). A substantial number of differentially expressed genes (DEGs) were identified between iCas9-H1 and ELTD1-deleted hESCs. Among these DEGs, mesoderm-associated genes such as MIXL1, EOMES, APLNR and BMP4 were downregulated, whereas HEP or hematopoiesis-associated genes including HOXA9, ITGB3 and RUNX1 were upregulated in ELTD1-deleted hESCs compared with iCas9-H1 (Fig. 4b). Furthermore, GSEA analysis revealed a significant enrichment of genes related to hematopoietic cell lineage in ELTD1-deleted cells compared with iCas9-H1 hESCs (Fig. 4c), consistent with the observed augmentation of HEPs and hematopoietic cells resulting from ELTD1 deletion.

To narrow down the genes for further analysis, we identified 439 common differential genes between iCas9-H1 and the two ELTD1-deleted hESCs (Fig. 4d) and performed KEGG analysis (Fig. 4e). We found these DEGs to be primarily enriched in the Wnt signaling pathway. Mounting evidence suggests that the Wnt signaling pathway plays a pivotal role in stem cell differentiation. Therefore, we analyzed changes in Wnt-associated genes at day 6, with or without ELTD1 deletion. Our findings revealed a notable downregulation of Wnt-associated genes in ELTD1-deleted cells (Fig. 4f). Apart from Wnt-associated genes, SOX17, a master regulator defining the hemogenic endothelium, which should be downregulated during EHT to promote commitment to blood cells, was also markedly downregulated in ELTD1-deleted cells^34^. Next, several Wnt-associated genes (TCF7L1, CCND1, BIRC5 and AXIN2) were selected for verification by qPCR (Fig. 4g), indicating that ELTD1 might function by mediating Wnt signals.

To delve deeper into the status of Wnt signaling at day 6 of hESC hematopoietic differentiation, we conducted western blotting analysis to measure the levels of phosphorylated β-catenin (at serine 33, 37 and 45; tyrosine 41), which are downstream mediators of the Wnt signaling^35^. As depicted in Fig. 4h, the phosphorylation level of β-catenin, reflecting β-catenin degradation, was higher in ELTD1-deleted hESCs compared with iCas9-H1 hESCs, indicating that Wnt signaling was inhibited by ELTD1 deletion. By contrast, ELTD1 overexpression led to reduced β-catenin phosphorylation, suggesting enhanced Wnt signaling activity. In summary, both bioinformatic analysis and functional studies revealed that ELTD1 deletion inhibits Wnt signaling during HEP generation.

### ELTD1 participates in HEP specification from hESCs via Wnt signaling

**Fig. 5:**
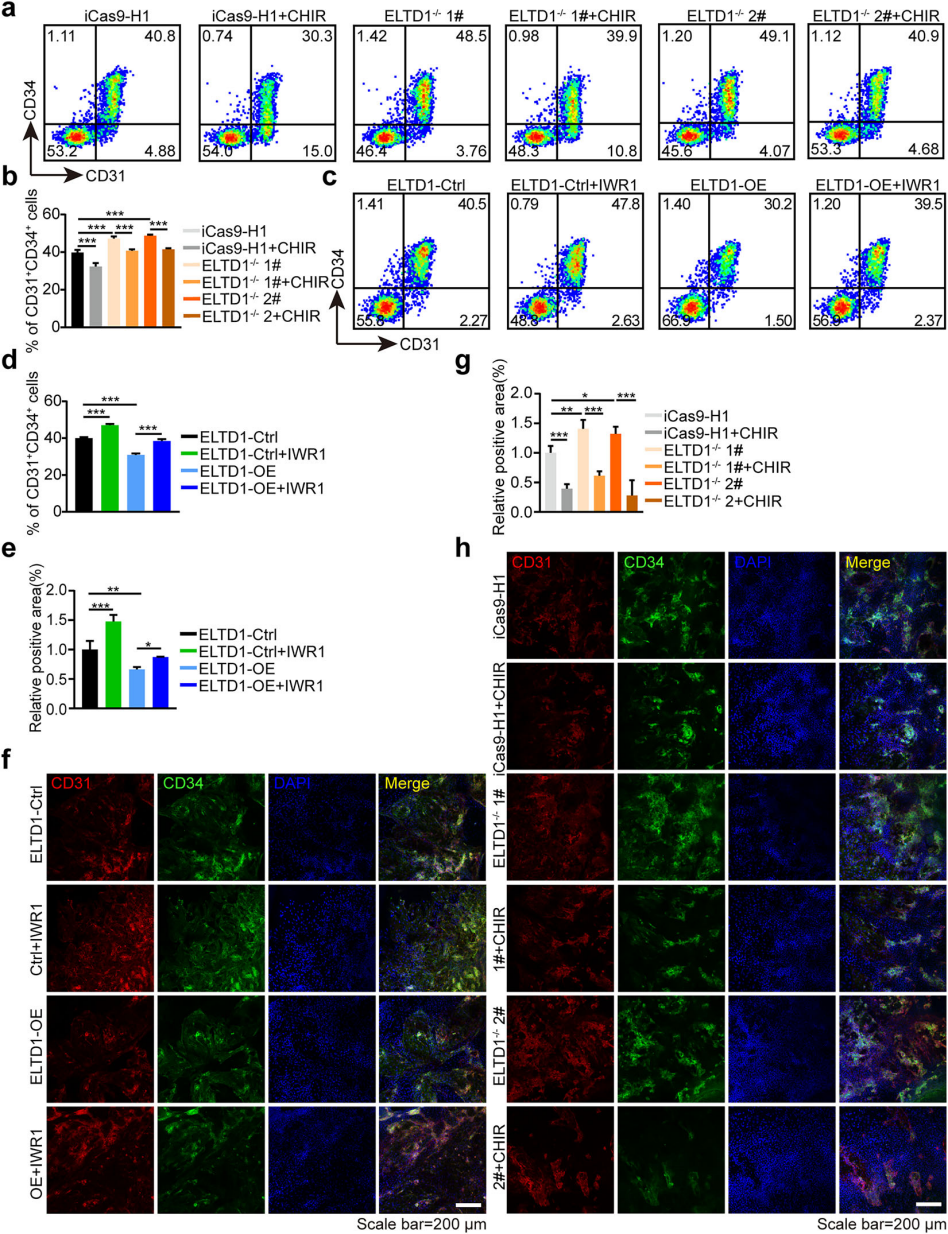
ELTD1 participates in HEP specification from hESCs via Wnt signaling. **a** Flow cytometry detection of CD31^+^CD34^+^ HEPs generated from iCas9-H1, ELTD1^−/−^ 1# and ELTD1^−/−^ 2# hESCs at day 6 of early hematopoietic differentiation after CHIR99021 treatment (2 μM) or not. **b** Statistical analysis of the flow cytometry data presented in **a** showing the percentage of CD31^+^CD34^+^ HEPs generated from iCas9-H1, ELTD1^−/−^ 1# and ELTD1^−/−^ 2# hESCs at day 6 of early hematopoietic differentiation. Results are shown as mean ± s.d.; *n* = 3. n.s., not significant, *P < 0.05, **P < 0.01, ***P < 0.001. **c** Flow cytometry detection of CD31^+^CD34^+^ HEPs generated from H1-ELTD1-Ctrl and H1-ELTD1-OE hESCs at day 6 of early hematopoietic differentiation after IWR1 treatment (2.5 μM) or not. **d** Statistical analysis of the flow cytometry data presented in **c** showing the percentage of CD31^+^CD34^+^ HEPs generated from H1-ELTD1-Ctrl and H1-ELTD1-OE hESCs at day 6 of early hematopoietic differentiation. Results are shown as mean ± s.d.; *n* = 3. n.s., not significant, *P < 0.05, **P < 0.01, ***P < 0.001. **e** Quantitative analysis of the immunostaining results presented in **f** Results are shown as mean ± s.d.; *n* = 3. n.s., not significant, *P < 0.05, **P < 0.01, ***P < 0.001. **f** Immunostaining of CD31^+^ (red) and CD34^+^ (green) generated from H1-ELTD1-Ctrl and H1-ELTD1-OE hESCs at day 6 of early hematopoietic differentiation after IWR1 treatment (2.5 μM) or not. Nuclei were counterstained with DAPI (blue). Scale bar, 200 μm. **g** Quantitative analysis of the immunostaining results presented in **h**. Results are shown as mean ± s.d.; *n* = 3. n.s., not significant, *P < 0.05, **P < 0.01, ***P < 0.001. **h** Immunostaining of CD31^+^ (red) and CD34^+^ (green) generated from iCas9-H1, ELTD1^−/−^ 1# and ELTD1^−/−^ 2# hESCs at day 6 of early hematopoietic differentiation after IWR1 treatment (2.5 μM) or not. Nuclei were counterstained with DAPI (blue). Scale bar, 200 μm.

**Fig. 6:**
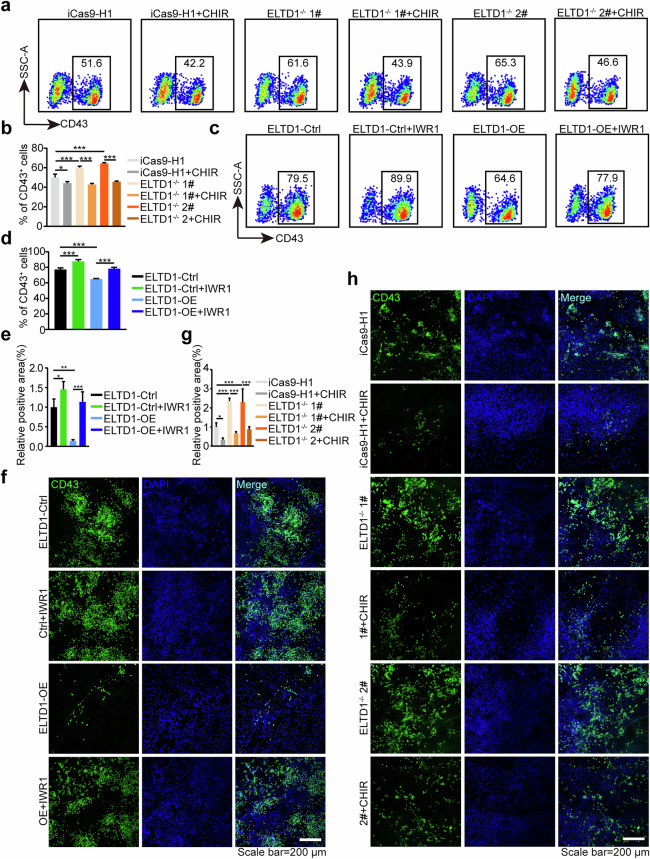
ELTD1 negatively regulates HEP specification from hESCs through Wnt signaling and modulates subsequent hematopoietic cell generation. **a** Flow cytometry detection of CD43^+^ hematopoietic cells generated from iCas9-H1, ELTD1^−/−^ 1# and ELTD1^−/−^ 2# hESCs at day 9 of early hematopoietic differentiation after CHIR99021 treatment (2 μM) or not. **b** Statistical analysis of the flow cytometry data presented in **a** showing the percentage of CD43^+^ hematopoietic cells generated from iCas9-H1, ELTD1^−/−^ 1# and ELTD1^−/−^ 2# hESCs at day 9 of early hematopoietic differentiation. Results are shown as mean ± s.d.; *n* = 3. n.s., not significant, *P < 0.05, **P < 0.01, ***P < 0.001. **c** Flow cytometry detection of CD43^+^ hematopoietic cells generated from H1-ELTD1-Ctrl and H1-ELTD1-OE hESCs at day 9 of early hematopoietic differentiation after IWR1 treatment (2.5 μM) or not. **d** Statistical analysis of the flow cytometry data presented in **c** showing the percentage of CD43^+^ hematopoietic cells generated from H1-ELTD1-Ctrl and H1-ELTD1-OE hESCs at day 9 of early hematopoietic differentiation. Results are shown as mean ± s.d.; *n* = 3. n.s., not significant, *P < 0.05, **P < 0.01, ***P < 0.001. **e** Quantitative analysis of the immunostaining results presented in **f**. Results are shown as mean ± s.d.; *n* = 3. n.s., not significant, *P < 0.05, **P < 0.01, ***P < 0.001. **f** Immunostaining of CD43^+^ (green) generated from H1-ELTD1-Ctrl and H1-ELTD1-OE hESCs at day 9 of early hematopoietic differentiation after IWR1 treatment (2.5 μM) or not. Nuclei were counterstained with DAPI (blue). Scale bar, 200 μm. **g** Quantitative analysis of the immunostaining results presented in **h**. Results are shown as mean ± s.d.; *n* = 3. n.s., not significant, *P < 0.05, **P < 0.01, ***P < 0.001. **h** Immunostaining of CD43^+^ (green) generated from iCas9-H1, ELTD1^−/−^ 1# and ELTD1^−/−^ 2# hESCs at day 9 of early hematopoietic differentiation after IWR1 treatment (2.5 μM) or not. Nuclei were counterstained with DAPI (blue). Scale bar, 200 μm.

As the above results showed that ELTD1 deletion inhibits Wnt signaling during HEP generation, we next assessed the relationship between ELTD1 and Wnt signaling. To determine whether the downregulation of Wnt signaling is essential for the improved early hematopoietic differentiation resulting from ELTD1 deletion in hESCs, we treated ELTD1^−/−^ 1# and ELTD1^−/−^ 2# hESCs with the Wnt agonist CHIR99021^36^. Based on the previous results that ELTD1 functions from mesoderm induction to HEP specification, we thus added CHIR99021 from days 3 to 6 for subsequent studies. As anticipated, the administration of CHIR99021 inhibited the generation of CD31^+^CD34^+^ HEPs at day 6 (Fig. 5a, b) compared with ELTD1 deletion alone, as validated by flow cytometry. To further validate these results, we then treated ELTD1-overexpressing hESCs with or without the Wnt antagonist IWR1^37^ from day 3 to 6 of differentiation. As depicted in Fig. 5c, d, ELTD1 overexpression robustly suppressed the proportion of CD31^+^CD34^+^ HEPs at day 6 but was reversed by treatment with the Wnt antagonist IWR1. These findings were further corroborated by immunofluorescence staining analysis (Fig. 5e–h). Consistent with these results, the proportion of CD43^+^ hematopoietic cells derived from HEPs at day 9 was significantly reduced after CHIR99021 treatment in the ELTD1 deletion group (Fig. 6a, b). Similarly, the addition of IWR1 markedly reversed the decline in CD43^+^ hematopoietic cells caused by ELTD1 overexpression (Fig. 6c, d). These findings were further supported by immunofluorescence staining experiments, which yielded consistent results (Fig. 6e–h). Furthermore, treatment with an alternative Wnt agonist (SKL2001) (Supplementary Fig. 9a, b and 9e, f) or Wnt antagonist (MSAB) (Supplementary Fig. 9c, d and 9g, h) from days 3 to 6 of differentiation produced identical outcomes. Collectively, these observations suggest that ELTD1 act as an upstream mediator of Wnt signaling in the regulation of HEP specification.

### ELTD1 regulates Wnt signaling via the HPIP–LEF1 axis to modulate HEP generation

**Fig. 7:**
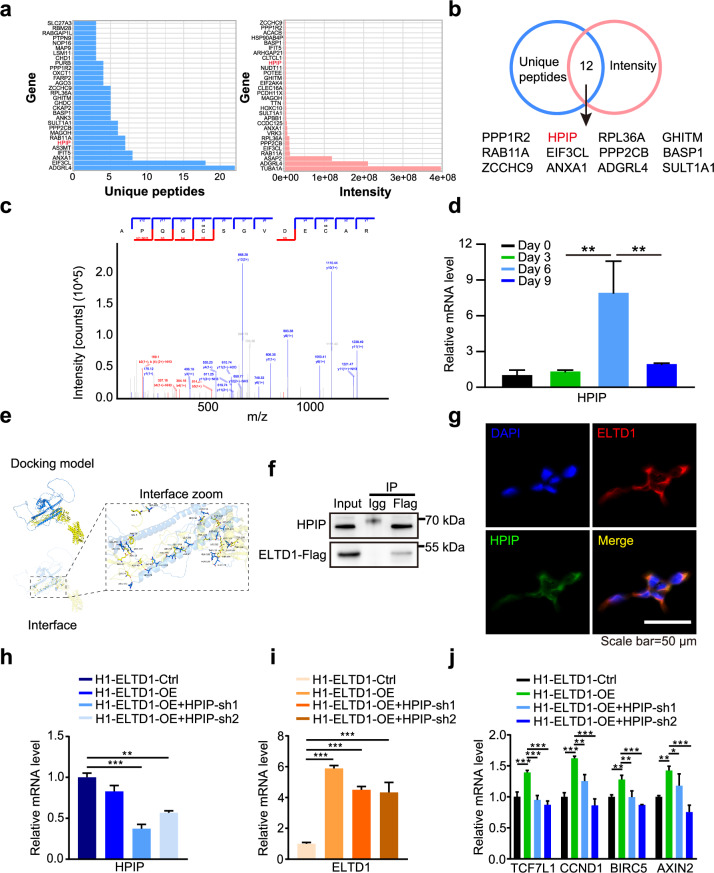
ELTD1 regulate HEP generation via HPIP. **a** Identification of proteins that potentially interact with ELTD1 through mass spectrometry analysis. **b** A schematic representation of the strategy used to screen and validate potential protein interactions with ELTD1. **c** Detailed mass spectrometry analysis of HPIP protein fragments. The secondary mass spectrum of HPIP is displayed, highlighting the key peptides used for protein identification. **d** Time course analysis of HPIP mRNA level with qPCR during hESC hematopoietic differentiation. Results are shown as mean ± s.d.; *n* = 3. n.s., not significant, *P < 0.05, **P < 0.01, ***P < 0.001. **e** A surface diagram of the docking model and their interfacing residues between ELTD1 and HPIP protein (ELTD1, yellow; HPIP, blue; hydrogen bond interaction, dotted line). **f** Co-IP analysis confirming the interaction between ELTD1 and HPIP proteins. **g** Immunofluorescence analysis was performed to visualize the colocalization of HPIP (green) and ELTD1 (red) in 293T cells. Nuclei were counterstained with DAPI (blue). Scale bar, 50 μm. **h** The mRNA levels of HPIP at day 6 of early hematopoietic differentiation in H1-ELTD1-Ctrl and H1-ELTD1-OE with or without HPIP knockdown. Results are shown as mean ± s.d.; *n* = 3. n.s., not significant, *P < 0.05, **P < 0.01, ***P < 0.001. **i** The mRNA levels of ELTD1 at day 6 of early hematopoietic differentiation in H1-ELTD1-Ctrl and H1-ELTD1-OE with or without HPIP knockdown. Results are shown as mean ± s.d.; *n* = 3. n.s., not significant, *P < 0.05, **P < 0.01, ***P < 0.001. **j** The mRNA level of representative Wnt signaling genes at day 6 of early hematopoietic differentiation in H1-ELTD1-Ctrl and H1-ELTD1-OE with or without HPIP knockdown. Results are shown as mean ± s.d.; *n* = 3. n.s., not significant, *P < 0.05, **P < 0.01, ***P < 0.001.

To investigate how ELTD1 regulates Wnt signaling pathway, we used co-IP coupled with mass spectrometry to identify its potential interaction partners. We screened the top 30 proteins based on the unique peptides and intensity analysis, respectively (Fig. 7a). Following our screening strategy, we identified 12 overlapped proteins that may interact with ELTD1 (Fig. 7b). Among them, HPIP (hematopoietic PBX1-interacting protein) attracted our attention (Fig. 7c) and was chosen for validation because it has been reported that HPIP deficiency could impair Wnt signaling in osteoarthritis (OA) chondrocytes^38^. Moreover, its dynamic expression pattern during hematopoietic differentiation is similar to that of ELTD1, with notable upregulation observed from day 3 to day 6 (Fig. 7d). To confirm this relationship, we subsequently conducted rigid protein–protein docking between ELTD1 and HPIP. As shown in Fig. 7e, ELTD1 and HPIP formed hydrogen bonds through amino acid residue sites such as ASN 180-THR 220 and GLN 181-THR 379, revealing that proteins ELTD1 and HPIP formed a stable protein docking model. Furthermore, we confirmed the formation of the ELTD1–HPIP complex through co-IP assays (Fig. 7f) and determined the subcellular localization of ELTD1 and HPIP using immunofluorescence staining. We found that ELTD1 and HPIP were primarily co-localized in the region where the cytoskeleton interacts with the cell membrane (Fig. 7g). These results suggested that ELTD1 might regulate Wnt signaling through interaction with HPIP.

To investigate whether HPIP is required for ELTD1-mediated expression of Wnt target genes, we knocked down HPIP in ELTD1-overexpressing hESCs and performed hematopoietic differentiation. The results revealed that ELTD1 overexpression increased the expression of Wnt targets (TCF7L1, CCND1, BIRC5 and AXIN2), whereas knockdown of HPIP abolished the ability of ELTD1 to modulate Wnt target production (Fig.7h–j), indicating the importance of HPIP in ELTD1-mediated regulation of Wnt target gene expression.

**Fig. 8:**
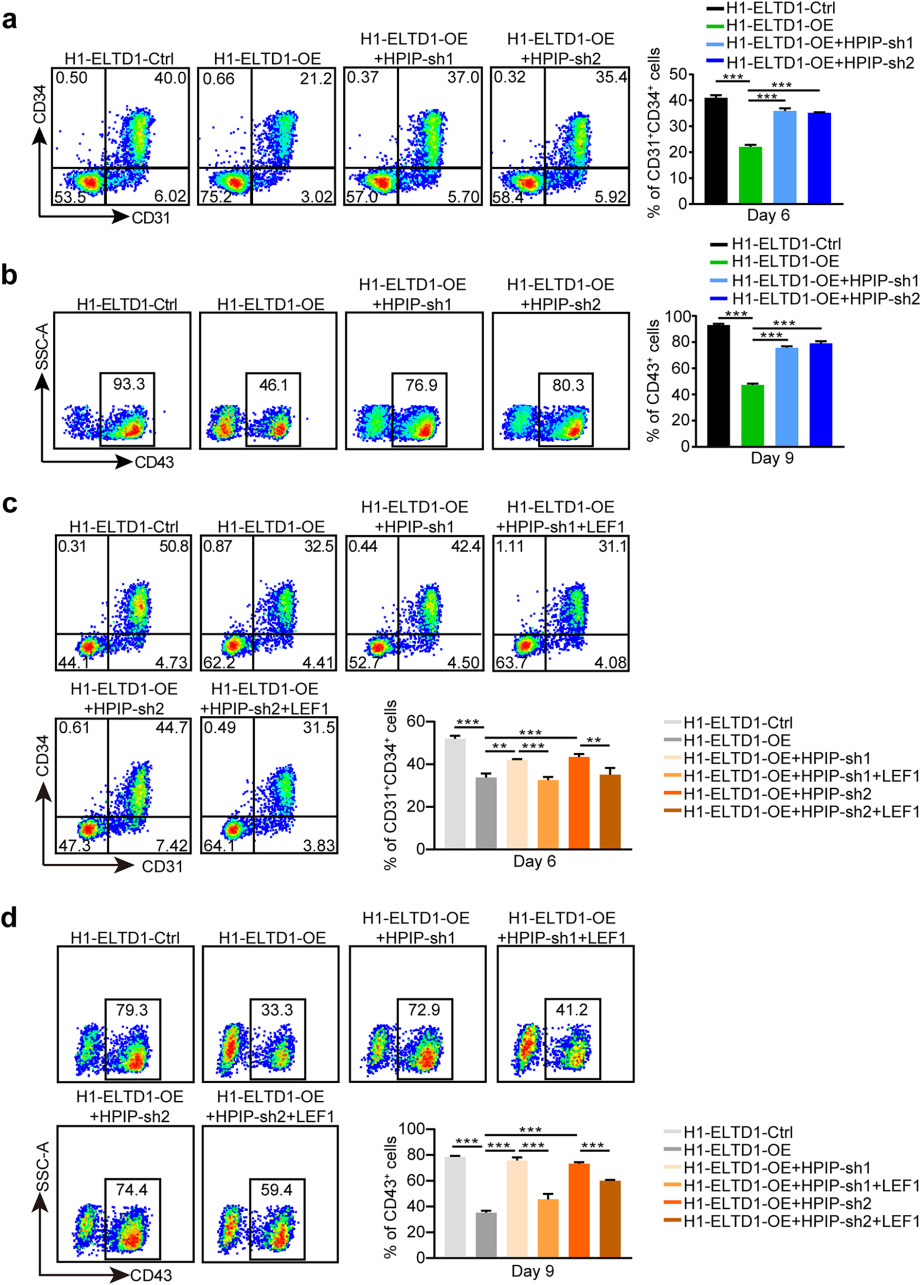
ELTD1 regulates Wnt signaling via the HPIP–LEF1 axis to modulate HEP generation. **a** The percentage of CD31^+^CD34^+^ HEPs at day 6 derived from H1-ELTD1-Ctrl and H1-ELTD1-OE with or without HPIP knockdown detected by flow cytometry. Results are shown as mean ± s.d.; *n* = 3. n.s., not significant, *P < 0.05, **P < 0.01, ***P < 0.001. **b** The percentage of CD43^+^ hematopoietic cells at day 9 derived from H1-ELTD1-Ctrl and H1-ELTD1-OE with or without HPIP knockdown detected by flow cytometry. Results are shown as mean ± s.d.; *n* = 3. n.s., not significant, *P < 0.05, **P < 0.01, ***P < 0.001. **c** The percentage of CD31^+^CD34^+^ HEPs at day 6 derived from H1-ELTD1-Ctrl and H1-ELTD1-OE cells with or without HPIP knockdown and LEF1 overexpression, detected by flow cytometry. Results are shown as mean ± s.d.; *n* = 3. n.s., not significant, *P < 0.05, **P < 0.01, ***P < 0.001. **d** The percentage of CD43^+^ hematopoietic cells at day 9 derived from H1-ELTD1-Ctrl and H1-ELTD1-OE with or without HPIP knockdown and LEF1 overexpression, detected by flow cytometry. Results are shown as mean ± s.d.; *n* = 3. n.s., not significant, *P < 0.05, **P < 0.01, ***P < 0.001.

Next, we asked whether HPIP knockdown could rescue the defects caused by ELTD1 overexpression. Indeed, knockdown of HPIP could rescue the negative effects of ELTD1 overexpression in HEP induction at day 6 (Fig. 8a). Moreover, inhibition of HPIP could further rescue the impaired CD43^+^ hematopoietic cells generation caused by ELTD1 overexpression at day 9 (Fig. 8b). Finally, we explored how ELTD1–HPIP regulates Wnt signaling. A prior study revealed that HPIP primarily mediates its biological effects by interacting with lymphoid enhancer binding factor 1 (LEF1) to activate Wnt signaling^38^. LEF1, a sequence-specific DNA-binding protein, is expressed in pre-B and T lymphocytes of adult mice, as well as in various embryonic tissues^39^. Therefore, we hypothesized that ELTD1–HPIP might promote Wnt signaling activation through LEF1 to mediate hematopoiesis. We overexpressed LEF1 in the aforementioned experiments and used flow cytometry to determine whether it could suppress the phenotypes induced by HPIP knockdown. As shown in Fig. 8c, overexpression of LEF1 attenuated the increase in the proportion of CD31^+^CD34^+^ HEPs at day 6 that was induced by HPIP knockdown in ELTD1-overexpressing hESCs. Consistent with these findings, LEF1 overexpression also inhibited the generation of CD43^+^ hematopoietic cells at day 9 (Fig. 8d). Taken together, these findings demonstrate that ELTD1 regulates the Wnt signaling pathway through the HPIP–LEF1 axis to modulate the generation of HEPs during hematopoietic differentiation of hESCs.

## Discussion

Elucidating the novel mechanism that regulates the formation of the HEP and its transition to a hematopoietic fate is important for us to accurately understand hematopoietic development in the early embryo and ultimately generate functional HSCs from pluripotent stem cells in vitro. In this study, we identified ELTD1 as a potential regulator of HEPs in early hESC hematopoietic differentiation and revealed its fundamental role in hematopoiesis. Suppression of ELTD1 remarkably increased the generation of HEPs, thereby enhancing hematopoietic differentiation in vitro. Mechanistically, we found that ELTD1 modulates the generation of HEPs by regulating the Wnt signaling pathway via the HPIP–LEF1 axis. Our results reveal a new mechanism for human hematopoiesis and may provide fresh ideas for efficient production of blood cells from hESCs in the fields of developmental biology and regenerative medicine.

As discovered in 2001 by Nechiporuk et al.^40^, ELTD1 is an orphan member of the GPCR superfamily, which is also known as ADGRL4. Since its discovery, ELTD1 has been found to participate in cardiac hypertrophy, renal cancer, ovarian cancer, colorectal cancer and glioblastoma^11,12,41–43^. However, the role of ELTD1 in early human hematopoietic development remains elusive. In this study, we analyzed the contribution of ELTD1 to human embryonic hematopoietic differentiation using H1 hESCs as a model. We analyzed the dynamic expression of ELTD1 during hematopoietic differentiation by using a hESC hematopoietic differentiation system in vitro. Our analysis indicated that the expression of ELTD1 peaked at day 6, which is in parallel with the expression of HEP-related genes. We further indicated that ELTD1 is specifically expressed in CD31^+^CD34^+^ HEPs rather than in other cell populations. Therefore, we speculated that ELTD1 may participate in HEP generation of human hematopoiesis. Subsequent functional assays confirmed our hypothesis, showing that ELTD1 inhibition increased the generation of HEPs rather than mesoderm or hematopoietic cell production, thus confirming its critical role in HEP differentiation.

Interestingly, in this study, we found that ELTD1 expression gradually increased during early hematopoietic differentiation, paralleling the emergence of the HEP population. However, its effect on HEPs was negative in loss-of-function studies. In both ELTD1-knockdown and ELTD1-deleted H1 hESCs, there was an increase in the proportion of CD31^+^CD34^+^ HEPs at day 6 of early hematopoietic differentiation, which was in contrast to its role identified in other endothelium studies. A previous study demonstrated that ELTD1 acts as a key player in tumor angiogenesis. Silencing ELTD1 drastically reduces tumor growth by impairing endothelial sprouting and vessel formation both in vitro and in vivo^44^. The observed variation between our study and previous research may be attributed to the diverse functions of ELTD1 across different stages and tissues during ontogeny. Hemogenic endothelium, a specialized subset of the endothelium with hematopoietic potential, is known to generate hematopoietic stem and progenitor cells through EHT^45,46^. Both hemogenic endothelium and vascular endothelium are developmentally related cells that share a common endothelial precursor^47,48^. Thus, downregulation of ELTD1 expression may be beneficial for the generation of hemopoiesis-biased endothelium, rather than vascular endothelium. In addition, Favara et al. showed that the HSC regulator KIT, which is expressed in hematopoietic stem and progenitor cells^49^ and required for hemogenic endothelium specification^50^, can be significantly upregulated at both the mRNA and protein levels by ELTD1 silencing^51^, which further supports our findings.

Mechanistically, we performed RNA-seq of differentiated cells at day 6 combined with rescue experiments. As expected, GSEA showed the enrichment of hematopoietic cell lineages-associated genes in ELTD1-deleted cells compared with iCas9-H1 cells, and KEGG analysis also displayed the same results. Expanding on the current understanding of ELTD1, we subsequently identified and confirmed Wnt signaling as the downstream target of ELTD1 that mediates the hematopoiesis. Accumulating evidence indicates that the Wnt signaling pathway is a conserved intercellular communication system crucial for stem cell proliferation, renewal and differentiation during embryonic development^52,53^. For example, inhibition of Wnt signaling by retinoic acid signaling is essential for HSC development^54^. However, the relationship between ELTD1 and Wnt signaling has not yet been explored. It has been widely reported that GPCRs can exert their functions via Wnt signaling^55,56^. By treating ELTD1-deleted hESCs with the Wnt agonist CHIR99021 or SKL2001, we found that the induction of CD31^+^CD34^+^ HEPs and subsequent production of CD43^+^ hematopoietic cells was dramatically inhibited compared with that in ELTD1-deleted hESCs. Similarly, the addition of the Wnt antagonist IWR1 or MSAB could rescue the defects of hematopoietic differentiation caused by ELTD1 overexpression. Collectively, these observations raise the interesting possibility that ELTD1 acts as an upstream of Wnt signaling and regulates early human hematopoiesis.

To investigate how ELTD1 regulates Wnt signaling during hESC hematopoietic differentiation, we performed protein mass spectrometry assay and found 12 candidates that may interact with ELTD1 based on the unique peptides and intensity analyses. Among these candidates, HPIP captured our interest owing to its deficiency, which has been shown to inhibit Wnt signaling in osteoarthritis chondrocytes^38^, suggesting that ELTD1 may mediate Wnt signaling through HPIP. HPIP is a scaffolding protein known to interact with various signaling proteins and participate in diverse cellular functions, including cell differentiation, proliferation and migration^57^. For instance, as a downstream target of GATA1, HPIP can promote erythroid differentiation via the PI3K–AKT pathway^58^. Based on the findings above, we speculate that HPIP is required for ELTD1 to activate the Wnt signaling pathway in hematopoiesis, thus warranting further investigation. In the present study, we verified the interaction between ELTD1 and HPIP protein. Subsequent analysis demonstrated that ELTD1 overexpression increased the expression of Wnt targets, whereas knockdown of HPIP inhibited the expression of Wnt targets caused by ELTD1 overexpression. Besides, we presented evidence that HPIP knockdown was able to rescue the defects of ELTD1 overexpression in HEP induction at day 6 and subsequent CD43^+^ hematopoietic cell generation at day 9. These results confirm our speculation that ELTD1 primarily exerts its biological function through interaction with HPIP to activate Wnt signaling in human hematopoiesis. Next, we sought to elucidate how the ELTD1–HPIP complex regulates Wnt signaling. It has been reported that HPIP interacts with transcription factors such as LEF1 to activate Wnt signaling. LEF1, also known as lymphoid enhancer factor 1, is a critical transcription factor involved in the Wnt–β-catenin signaling pathway. It belongs to the TCF–LEF family of proteins and plays a key role in regulating the expression of genes involved in cell proliferation, differentiation and survival^59,60^. Through overexpression of LEF1 in HPIP-knockdown hESCs and subsequent functional analyses, we identified LEF1 as a downstream factor in the ELTD1–HPIP axis that regulates HEP generation. Although the role of HPIP in modulating the Wnt signaling pathway through its interaction with LEF1 has been previously reported, our study establishes a functional link between ELTD1, HPIP, LEF1 and Wnt signaling, uncovering a novel regulatory mechanism that governs early hematopoiesis. Our work highlights the ELTD1–HPIP–LEF1–Wnt axis as a regulatory pathway influencing hematopoietic differentiation. However, several unresolved issues remain. For instance, the precise mechanism by which the ELTD1–HPIP complex regulates Wnt signaling via LEF1 requires further investigation. We hypothesize that ELTD1 may enhance HPIP’s ability to interact with LEF1 or stabilize the HPIP–LEF1 complex, thereby potentiating Wnt signaling. To address this, future experiments, such as co-IP and chromatin immunoprecipitation assays, will be conducted to elucidate the molecular interactions among ELTD1, HPIP and LEF1 in greater detail.

In summary, the findings of this study identify a pivotal role of ELTD1 in early hESC hematopoietic differentiation. Besides, we provide evidence that it performs this function through Wnt signaling and revealed a molecular network around ELTD1, HPIP, LEF1 and Wnt signaling in early hematopoiesis. This study offers insights into the function of ELTD1 in regulating hematopoietic specification in vitro and may offer clues for optimizing in vitro differentiation methods for generating HSCs for future clinical applications.

## Supplementary information

Supplementary Information

## References

[1] Copelan, E. A. Hematopoietic stem-cell transplantation. *N. Engl. J. Med* **354**, 1813–1826 (2006). 16641398 10.1056/NEJMra052638

[2] Murry, C. E. & Keller, G. Differentiation of embryonic stem cells to clinically relevant populations: lessons from embryonic development. *Cell* **132**, 661–680 (2008). 18295582 10.1016/j.cell.2008.02.008

[3] Chen, X. et al. Integrative epigenomic and transcriptomic analysis reveals the requirement of JUNB for hematopoietic fate induction. *Nat. Commun.* **13**, 3131 (2022). 35668082 10.1038/s41467-022-30789-4 PMC9170695

[4] Luo, Q. et al. Specific blood cells derived from pluripotent stem cells: an emerging field with great potential in clinical cell therapy. *Stem Cells Int* **2021**, 9919422 (2021). 34434242 10.1155/2021/9919422 PMC8380505

[5] Slukvin, I. I. Hematopoietic specification from human pluripotent stem cells: current advances and challenges toward de novo generation of hematopoietic stem cells. *Blood* **122**, 4035–4046 (2013). 24124087 10.1182/blood-2013-07-474825 PMC3862281

[6] Zhang, P. et al. G protein-coupled receptor 183 facilitates endothelial-to-hematopoietic transition via Notch1 inhibition. *Cell Res.* **25**, 1093–1107 (2015). 26358189 10.1038/cr.2015.109 PMC4650626

[7] Wang, Y. et al. LGR4, not LGR5, enhances hPSC hematopoiesis by facilitating mesoderm induction via TGF-β signaling activation. *Cell Rep.* **31**, 107600 (2020). 32375050 10.1016/j.celrep.2020.107600

[8] Solaimani, K. P. et al. Whole-transcriptome analysis of endothelial to hematopoietic stem cell transition reveals a requirement for Gpr56 in HSC generation. *J. Exp. Med.* **212**, 93–106 (2015). 25547674 10.1084/jem.20140767 PMC4291529

[9] Lancrin, C. et al. The haemangioblast generates haematopoietic cells through a haemogenic endothelium stage. *Nature* **457**, 892–895 (2009). 19182774 10.1038/nature07679 PMC2661201

[10] Li, H. et al. Biomechanical cues as master regulators of hematopoietic stem cell fate. *Cell. Mol. Life Sci.* **78**, 5881–5902 (2021). 34232331 10.1007/s00018-021-03882-y PMC8316214

[11] Favara, D. M., Banham, A. H. & Harris, A. L. A review of ELTD1, a pro-angiogenic adhesion GPCR. *Biochem. Soc. Trans.* **42**, 1658–1664 (2014). 25399586 10.1042/BST20140216

[12] Serban, F. et al. Epidermal growth factor, latrophilin, and seven transmembrane domain-containing protein 1 marker, a novel angiogenesis marker. *Onco Targets Ther.* **8**, 3767–3774 (2015). 26719704 10.2147/OTT.S93843 PMC4689259

[13] Mcquade, A. et al. Development and validation of a simplified method to generate human microglia from pluripotent stem cells. *Mol. Neurodegener.* **13**, 67 (2018). 30577865 10.1186/s13024-018-0297-x PMC6303871

[14] Luo, Q. et al. Generation of an ELTD1 knockout human embryonic stem cell line by the iCRISPR/Cas9 system. *Stem Cell Res.* **53**, 102350 (2021). 34087984 10.1016/j.scr.2021.102350

[15] Li, L. et al. Snakin-2 interacts with cytosolic glyceraldehyde-3-phosphate dehydrogenase 1 to inhibit sprout growth in potato tubers. *Horticult. Res.* **9**, uhab060 (2022). 10.1093/hr/uhab060 PMC8972991 35043182

[16] Wang, H. et al. MEIS1 regulates hemogenic endothelial generation, megakaryopoiesis, and thrombopoiesis in human pluripotent stem cells by targeting TAL1 and FLI1. *Stem Cell Rep.* **10**, 447–460 (2018). 10.1016/j.stemcr.2017.12.017 PMC5830947 29358086

[17] Zhao, H. & Choi, K. Single cell transcriptome dynamics from pluripotency to FLK1^+^ mesoderm. *Development* **146**, dev182097 (2019). 10.1242/dev.182097 PMC6918769 31740535

[18] Vodyanik, M. A. et al. A mesoderm-derived precursor for mesenchymal stem and endothelial cells. *Cell Stem Cell* **7**, 718–729 (2010). 21112566 10.1016/j.stem.2010.11.011 PMC3033587

[19] Vodyanik, M. A., Bork, J. A., Thomson, J. A. & Slukvin, I. I. Human embryonic stem cell-derived CD34^+^ cells: efficient production in the coculture with OP9 stromal cells and analysis of lymphohematopoietic potential. *Blood* **105**, 617–626 (2005). 15374881 10.1182/blood-2004-04-1649

[20] Huang, K. et al. Generation and analysis of GATA2(w/eGFP) human ESCs reveal ITGB3/CD61 as a reliable marker for defining hemogenic endothelial cells during hematopoiesis. *Stem Cell Rep.* **7**, 854–868 (2016). 10.1016/j.stemcr.2016.09.008 PMC5106517 27746115

[21] Vodyanik, M. A., Thomson, J. A. & Slukvin, I. I. Leukosialin (CD43) defines hematopoietic progenitors in human embryonic stem cell differentiation cultures. *Blood* **108**, 2095–2105 (2006). 16757688 10.1182/blood-2006-02-003327 PMC1895535

[22] Verfaillie, C. M. Adhesion receptors as regulators of the hematopoietic process. *Blood* **92**, 2609–2612 (1998). 9763542

[23] Lin, H. H. et al. Adhesion GPCRs in regulating immune responses and inflammation. *Adv. Immunol.* **136**, 163–201 (2017). 28950945 10.1016/bs.ai.2017.05.005

[24] Maglitto, A. et al. Unexpected redundancy of Gpr56 and Gpr97 during hematopoietic cell development and differentiation. *Blood Adv.* **5**, 829–842 (2021). 33560396 10.1182/bloodadvances.2020003693 PMC7876891

[25] Garcia-Alegria, E. et al. Early human hemogenic endothelium generates primitive and definitive hematopoiesis in vitro. *Stem Cell Rep.* **11**, 1061–1074 (2018). 10.1016/j.stemcr.2018.09.013 PMC6234921 30449319

[26] Zeng, Y. et al. Tracing the first hematopoietic stem cell generation in human embryo by single-cell RNA sequencing. *Cell Res.* **29**, 881–894 (2019). 31501518 10.1038/s41422-019-0228-6 PMC6888893

[27] Lilly, A. J. et al. Interplay between SOX7 and RUNX1 regulates hemogenic endothelial fate in the yolk sac. *Development* **143**, 4341–4351 (2016). 27802172 10.1242/dev.140970

[28] Zhou, Y. et al. Overexpression of GATA2 enhances development and maintenance of human embryonic stem cell-derived hematopoietic stem cell-like progenitors. *Stem Cell Rep.* **13**, 31–47 (2019). 10.1016/j.stemcr.2019.05.007 PMC6626852 31178416

[29] Wang, M. et al. MEIS2 regulates endothelial to hematopoietic transition of human embryonic stem cells by targeting TAL1. *Stem Cell Res. Ther.* **9**, 340 (2018). 30526668 10.1186/s13287-018-1074-z PMC6286587

[30] Chen, M. J., Yokomizo, T., Zeigler, B. M., Dzierzak, E. & Speck, N. A. Runx1 is required for the endothelial to haematopoietic cell transition but not thereafter. *Nature* **457**, 887–891 (2009). 19129762 10.1038/nature07619 PMC2744041

[31] Iacovino, M. et al. HoxA3 is an apical regulator of haemogenic endothelium. *Nat. Cell Biol.* **13**, 72–78 (2011). 21170035 10.1038/ncb2137 PMC3079247

[32] Ramos-Mejia, V. et al. HOXA9 promotes hematopoietic commitment of human embryonic stem cells. *Blood* **124**, 3065–3075 (2014). 25185710 10.1182/blood-2014-03-558825

[33] Gonzalez, F. et al. An iCRISPR platform for rapid, multiplexable, and inducible genome editing in human pluripotent stem cells. *Cell Stem Cell* **15**, 215–226 (2014). 24931489 10.1016/j.stem.2014.05.018 PMC4127112

[34] Nakajima-Takagi, Y. et al. Role of SOX17 in hematopoietic development from human embryonic stem cells. *Blood* **121**, 447–458 (2013). 23169777 10.1182/blood-2012-05-431403

[35] Clevers, H. & Nusse, R. Wnt/β-catenin signaling and disease. *Cell* **149**, 1192–1205 (2012). 22682243 10.1016/j.cell.2012.05.012

[36] Broda, T. R., Mccracken, K. W. & Wells, J. M. Generation of human antral and fundic gastric organoids from pluripotent stem cells. *Nat. Protoc.* **14**, 28–50 (2019). 30470820 10.1038/s41596-018-0080-z PMC7951181

[37] Martins-Neves, S. R. et al. IWR-1, a tankyrase inhibitor, attenuates Wnt/β-catenin signaling in cancer stem-like cells and inhibits in vivo the growth of a subcutaneous human osteosarcoma xenograft. *Cancer Lett.* **414**, 1–15 (2018). 29126913 10.1016/j.canlet.2017.11.004

[38] Ji, Q. et al. Hematopoietic PBX-interacting protein mediates cartilage degeneration during the pathogenesis of osteoarthritis. *Nat. Commun.* **10**, 313 (2019). 30659184 10.1038/s41467-018-08277-5 PMC6338798

[39] Elayyan, J. et al. LEF1-mediated MMP13 gene expression is repressed by SIRT1 in human chondrocytes. *FASEB J.* **31**, 3116–3125 (2017). 28389425 10.1096/fj.201601253R

[40] Nechiporuk, T., Urness, L. D. & Keating, M. T. ETL, a novel seven-transmembrane receptor that is developmentally regulated in the heart. ETL is a member of the secretin family and belongs to the epidermal growth factor-seven-transmembrane subfamily. *J. Biol. Chem.* **276**, 4150–4157 (2001). 11050079 10.1074/jbc.M004814200

[41] Xiao, J. et al. Augmented cardiac hypertrophy in response to pressure overload in mice lacking ELTD1. *PLoS ONE* **7**, e35779 (2012). 22606234 10.1371/journal.pone.0035779 PMC3350503

[42] Ziegler, J. et al. ELTD1, an effective anti-angiogenic target for gliomas: preclinical assessment in mouse GL261 and human G55 xenograft glioma models. *Neuro Oncol.* **19**, 175–185 (2017). 27416955 10.1093/neuonc/now147 PMC5464087

[43] Niinivirta, M. et al. Tumor endothelial ELTD1 as a predictive marker for treatment of renal cancer patients with sunitinib. *BMC Cancer* **20**, 339 (2020). 32321460 10.1186/s12885-020-06770-z PMC7179003

[44] Masiero, M. et al. A core human primary tumor angiogenesis signature identifies the endothelial orphan receptor ELTD1 as a key regulator of angiogenesis. *Cancer Cell* **24**, 229–241 (2013). 23871637 10.1016/j.ccr.2013.06.004 PMC3743050

[45] Marcelo, K. L., Goldie, L. C. & Hirschi, K. K. Regulation of endothelial cell differentiation and specification. *Circ. Res.* **112**, 1272–1287 (2013). 23620236 10.1161/CIRCRESAHA.113.300506 PMC3768127

[46] Gritz, E. & Hirschi, K. K. Specification and function of hemogenic endothelium during embryogenesis. *Cell. Mol. Life Sci.* **73**, 1547–1567 (2016). 26849156 10.1007/s00018-016-2134-0 PMC4805691

[47] Kissa, K. & Herbomel, P. Blood stem cells emerge from aortic endothelium by a novel type of cell transition. *Nature* **464**, 112–115 (2010). 20154732 10.1038/nature08761

[48] Kennedy, M. et al. A common precursor for primitive erythropoiesis and definitive haematopoiesis. *Nature* **386**, 488–493 (1997). 9087406 10.1038/386488a0

[49] Edling, C. E. & Hallberg, B. c-Kit-a hematopoietic cell essential receptor tyrosine kinase. *Int J. Biochem Cell Biol.* **39**, 1995–1998 (2007). 17350321 10.1016/j.biocel.2006.12.005

[50] Marcelo, K. L. et al. Hemogenic endothelial cell specification requires c-Kit, Notch signaling, and p27-mediated cell-cycle control. *Dev. Cell* **27**, 504–515 (2013). 24331925 10.1016/j.devcel.2013.11.004 PMC3994666

[51] Favara, D. M. et al. ADGRL4/ELTD1 silencing in endothelial cells induces ACLY and SLC25A1 and alters the cellular metabolic profile. *Metabolites* **9**, 287 (2019). 10.3390/metabo9120287 PMC6950702 31775252

[52] Nusse, R. & Clevers, H. Wnt/β-catenin signaling, disease, and emerging therapeutic modalities. *Cell* **169**, 985–999 (2017). 28575679 10.1016/j.cell.2017.05.016

[53] Steinhart, Z. & Angers, S. Wnt signaling in development and tissue homeostasis. *Development* **145**, dev146589 (2018). 10.1242/dev.146589 29884654

[54] Chanda, B., Ditadi, A., Iscove, N. N. & Keller, G. Retinoic acid signaling is essential for embryonic hematopoietic stem cell development. *Cell* **155**, 215–227 (2013). 24074870 10.1016/j.cell.2013.08.055

[55] Li, B. I. et al. The orphan GPCR, Gpr161, regulates the retinoic acid and canonical Wnt pathways during neurulation. *Dev. Biol.* **402**, 17–31 (2015). 25753732 10.1016/j.ydbio.2015.02.007

[56] Dietrich, P. A. et al. GPR84 sustains aberrant β-catenin signaling in leukemic stem cells for maintenance of MLL leukemogenesis. *Blood* **124**, 3284–3294 (2014). 25293777 10.1182/blood-2013-10-532523

[57] Penugurti, V. et al. HPIP protooncogene differentially regulates metabolic adaptation and cell fate in breast cancer cells under glucose stress via AMPK and RNF2 dependent pathways. *Cancer Lett.* **518**, 243–255 (2021). 34302919 10.1016/j.canlet.2021.07.027

[58] Manavathi, B. et al. Functional regulation of pre-B-cell leukemia homeobox interacting protein 1 (PBXIP1/HPIP) in erythroid differentiation. *J. Biol. Chem.* **287**, 5600–5614 (2012). 22187427 10.1074/jbc.M111.289843 PMC3285334

[59] Luo, Y. et al. The osteogenic differentiation of human adipose-derived stem cells is regulated through the let-7i-3p/LEF1/β-catenin axis under cyclic strain. *Stem Cell Res Ther.* **10**, 339 (2019). 31753039 10.1186/s13287-019-1470-z PMC6873506

[60] Hao, Y. et al. Induction of LEF1 by MYC activates the WNT pathway and maintains cell proliferation. *Cell Commun. Signal* **17**, 129 (2019). 31623618 10.1186/s12964-019-0444-1 PMC6798382

