# Supplementary material for: ELTD1 inhibits differentiation of hemogenic endothelium progenitors from human embryonic stem cells through the HPIP–Wnt pathway
Source: Exp Mol Med. 2025 Jun 2;57(6):1216–31. doi: 10.1038/s12276-025-01473-6 (PMC12229684; doi:10.1038/s12276-025-01473-6)
Supplement: Supplementary file 1 — Supplementary Information [file 12276_2025_1473_MOESM1_ESM.pdf]

# Supplementary Figure 1

**a**

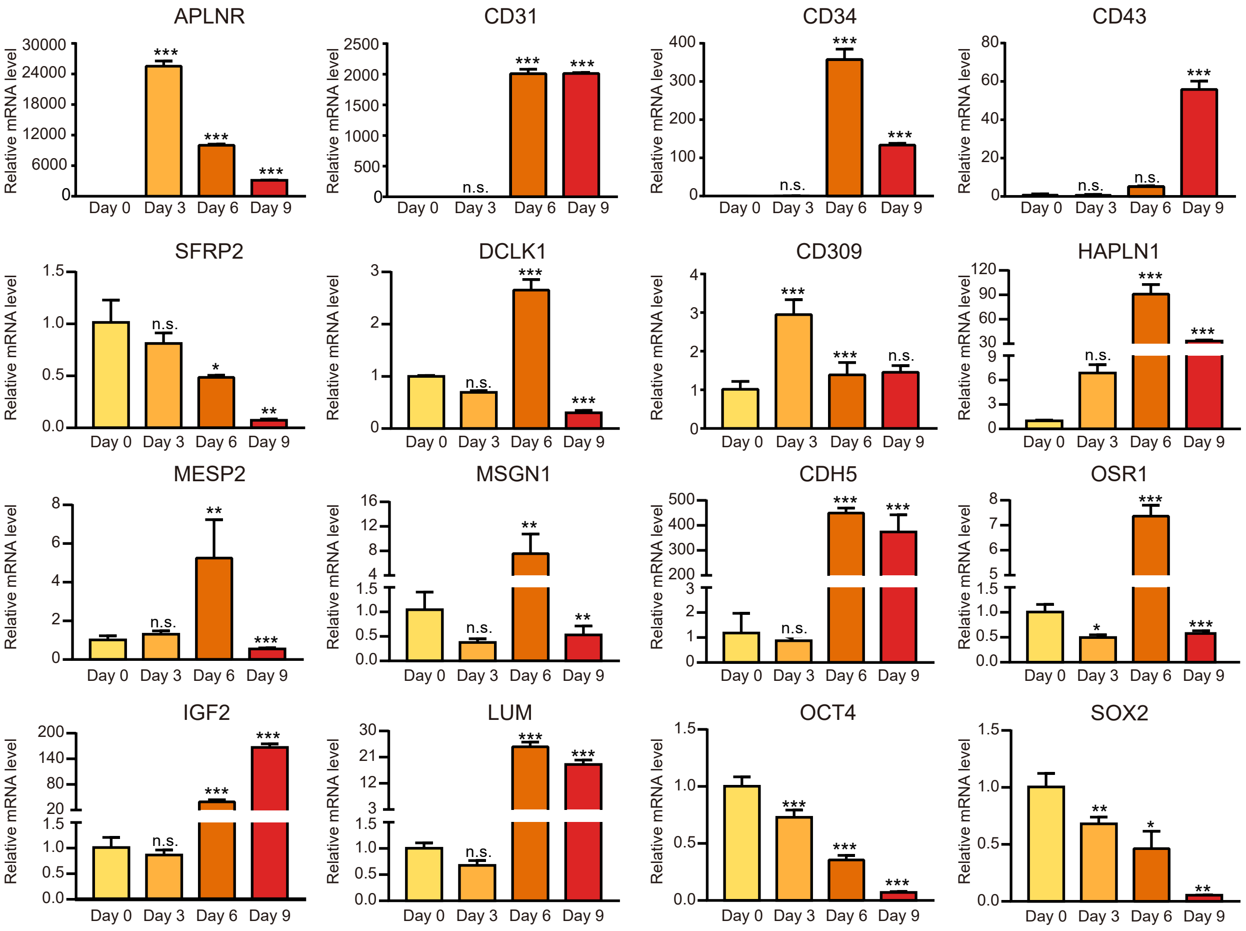


**b**

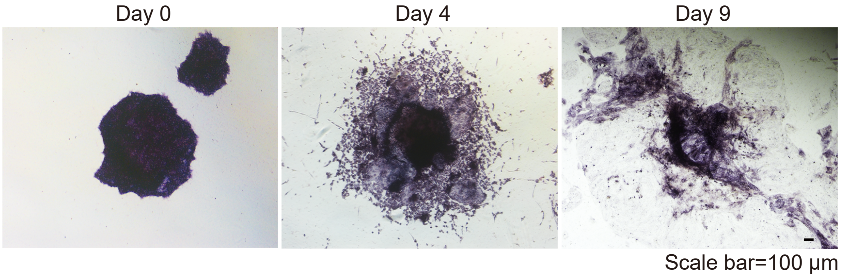

# Supplementary Figure 2

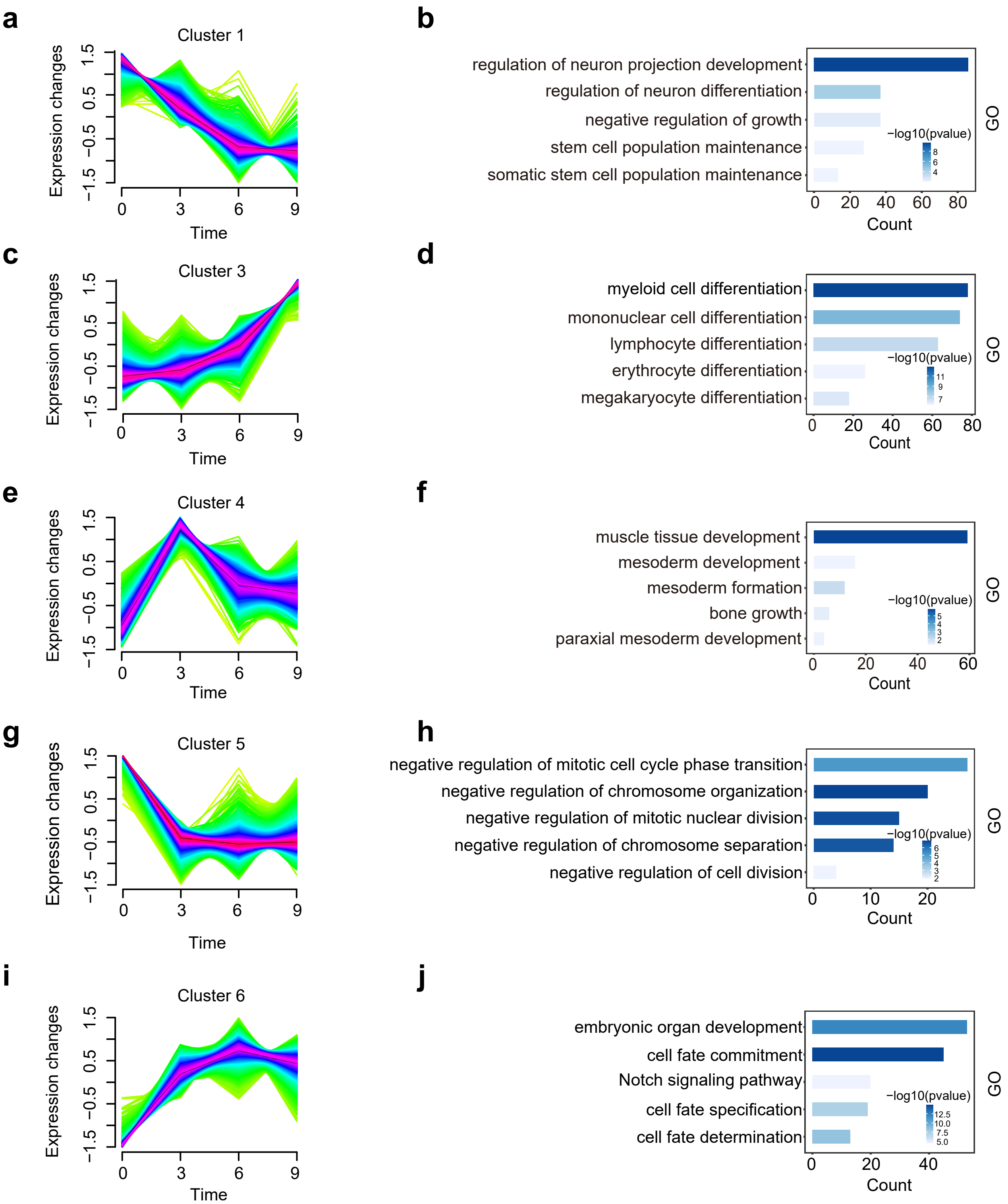

# Supplementary Figure 3

**a**

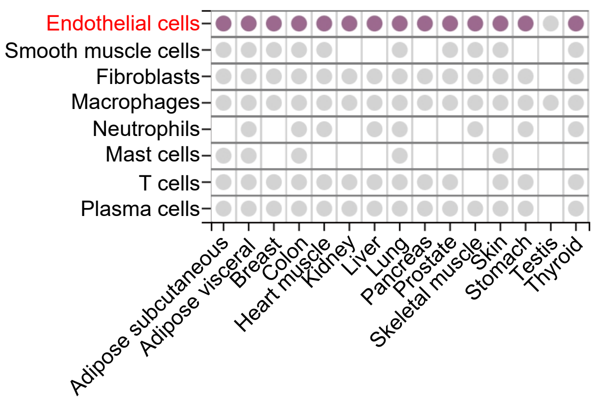


**b**

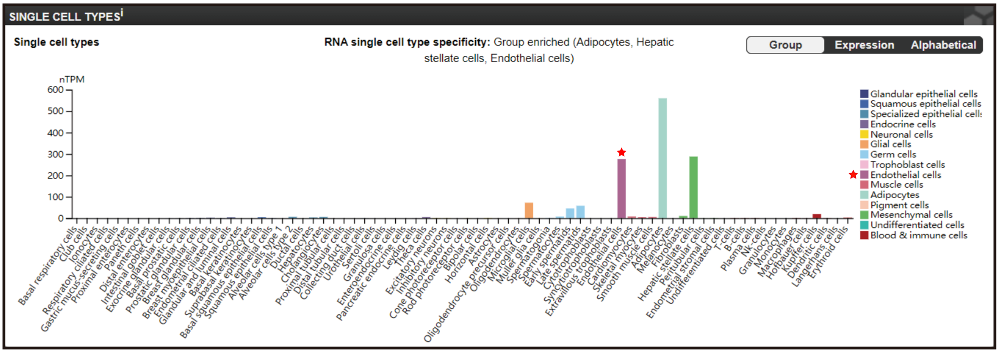


**c**

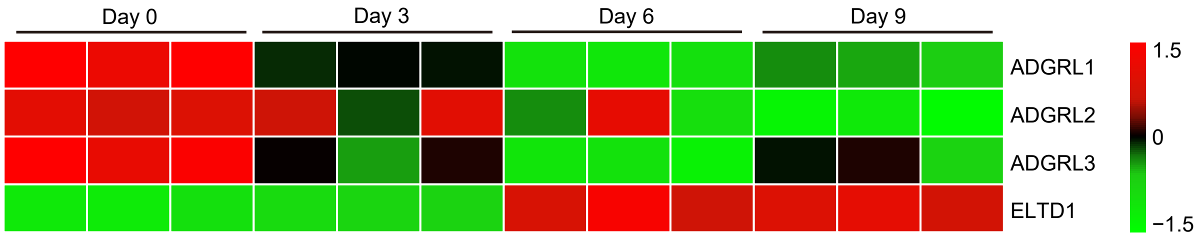

# Supplementary Figure 4

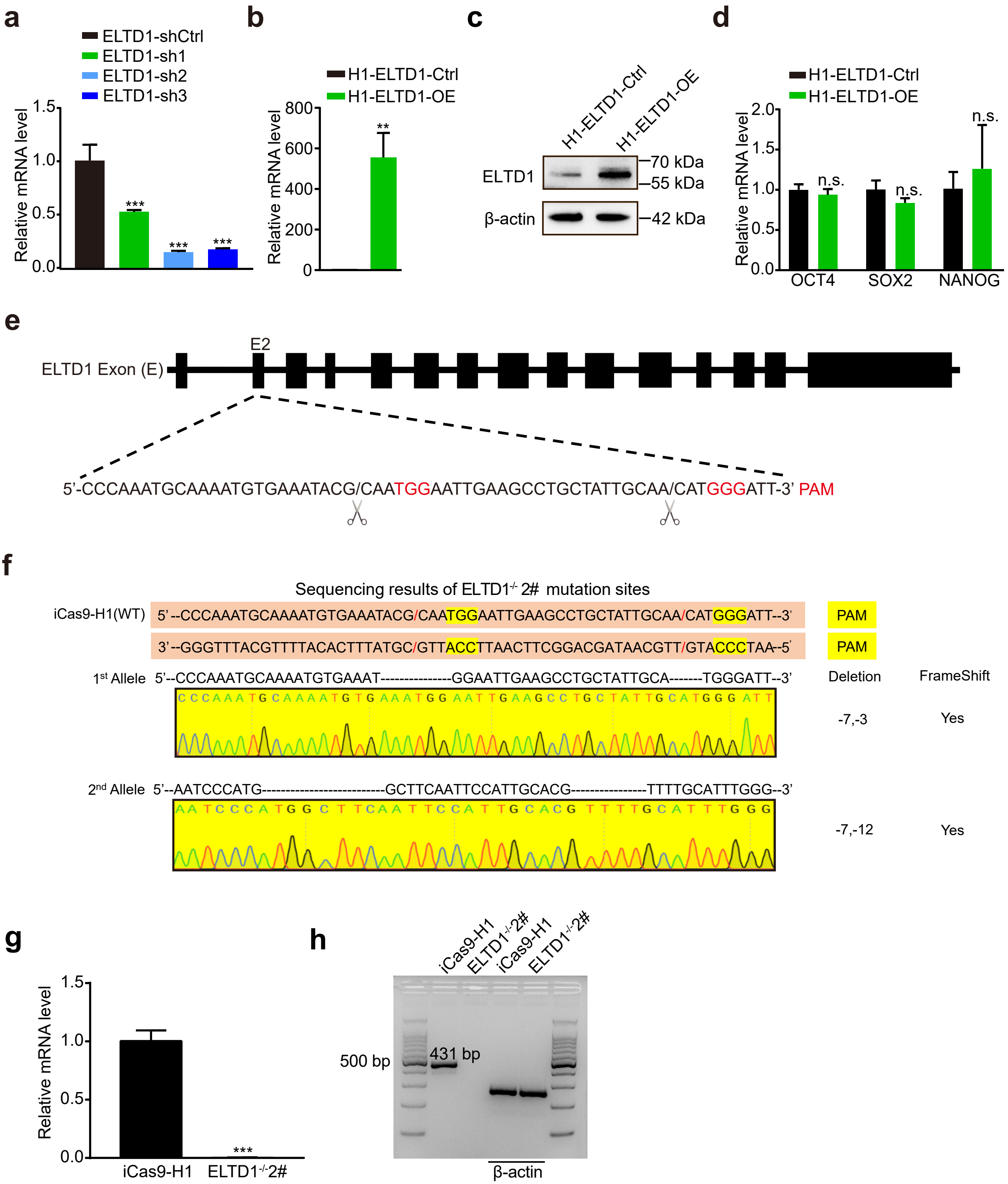

## Supplementary Figure 5

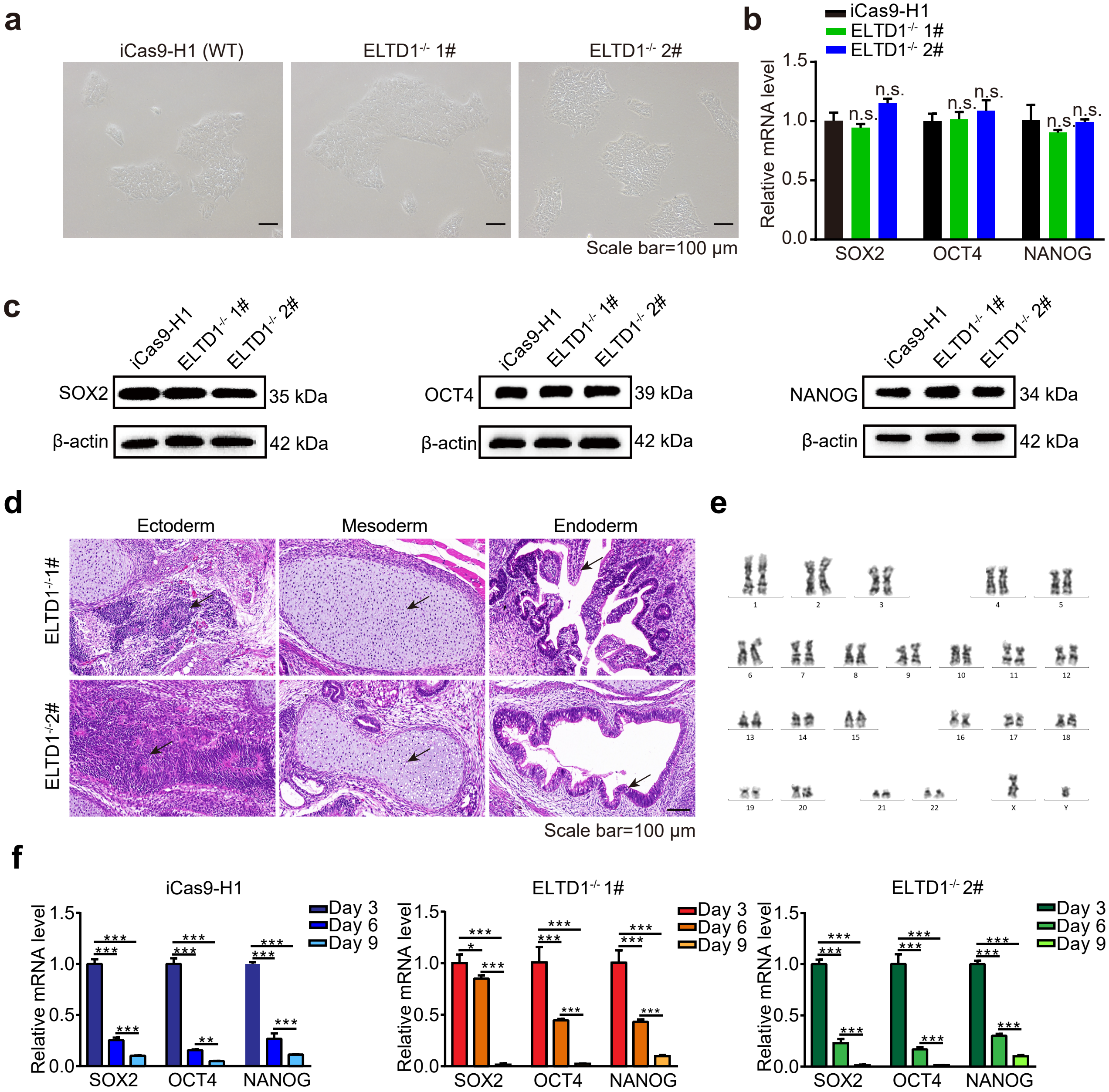

# Supplementary Figure 6

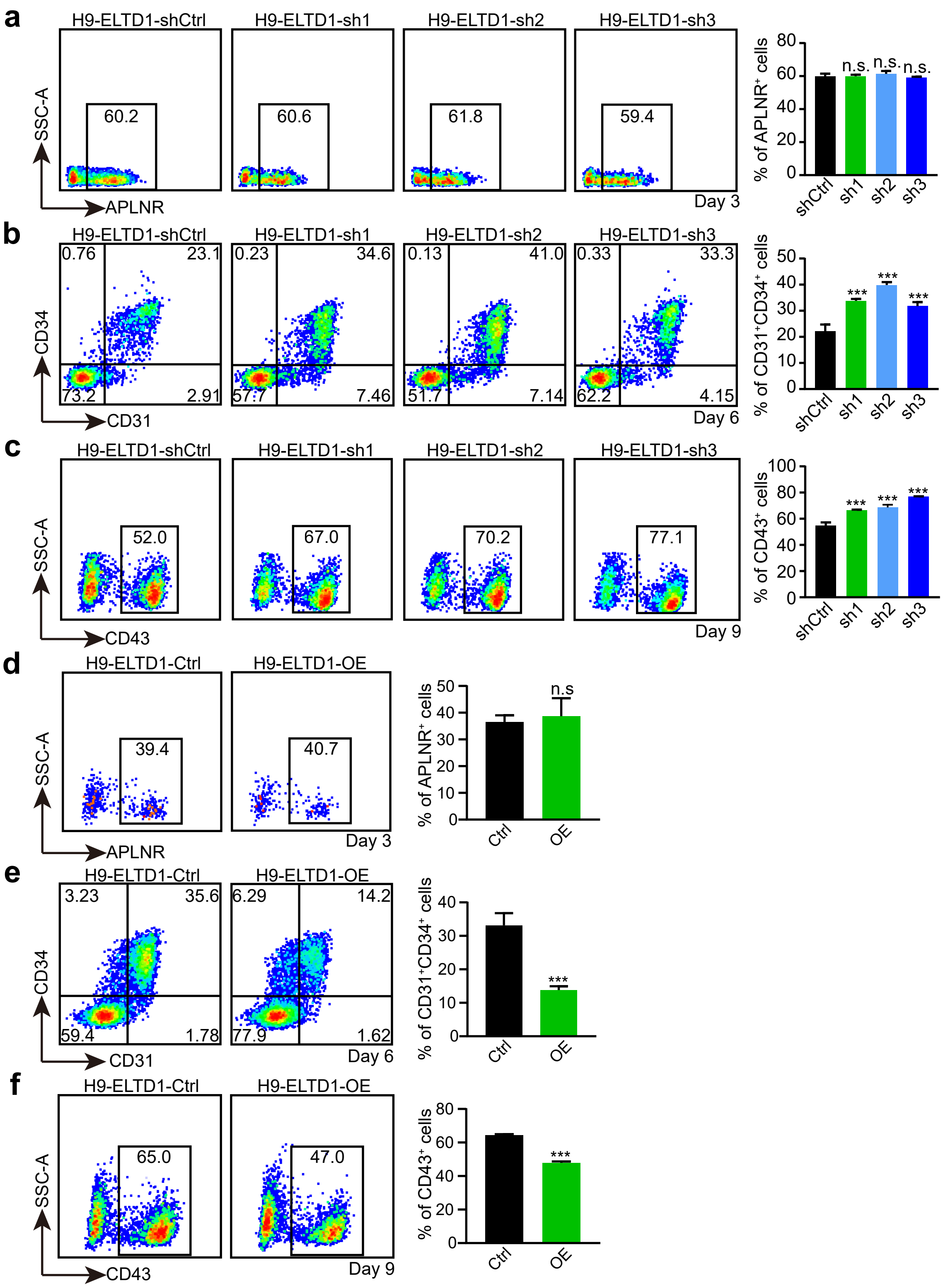

# Supplementary Figure 7

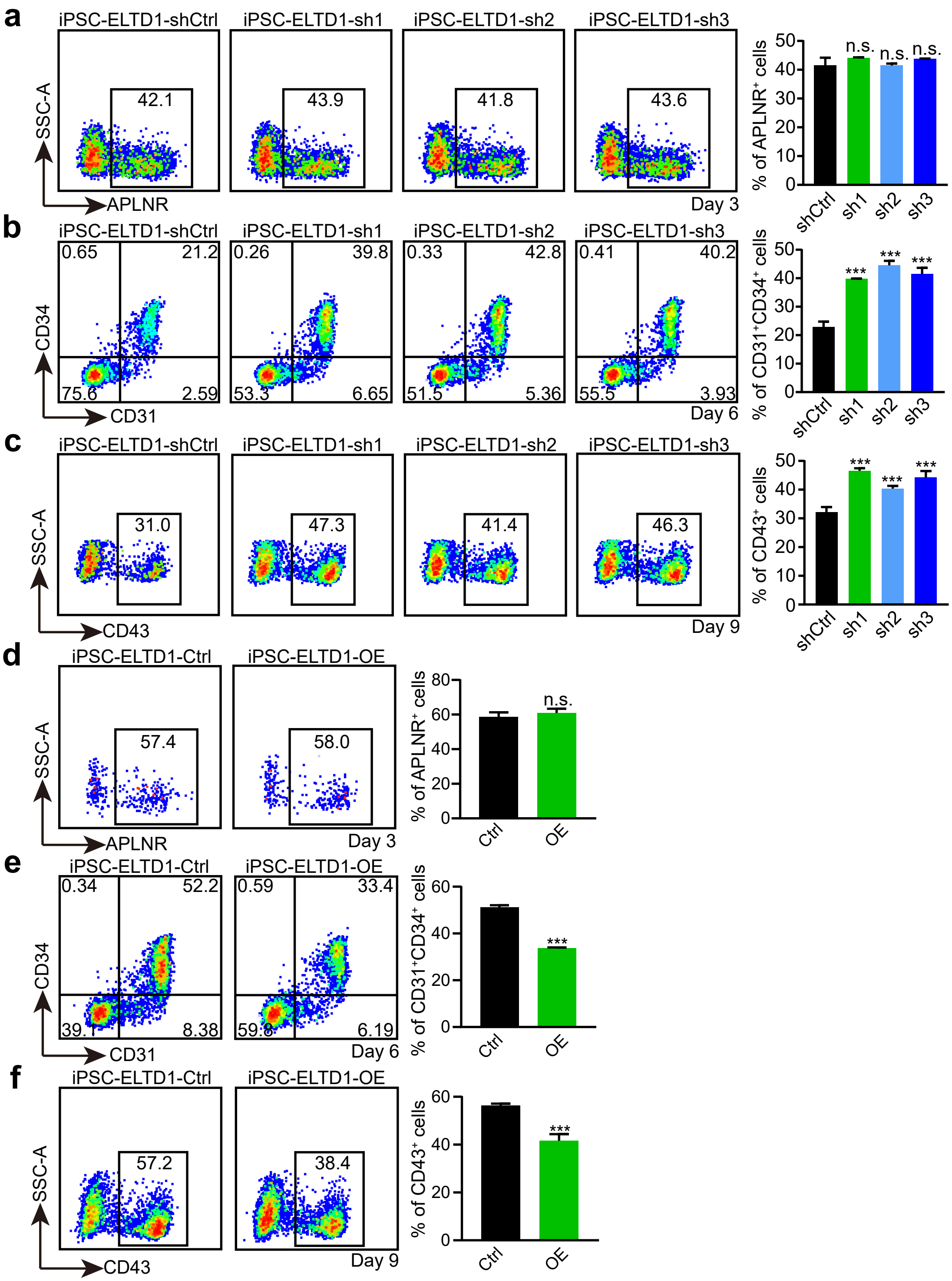

# Supplementary Figure 8

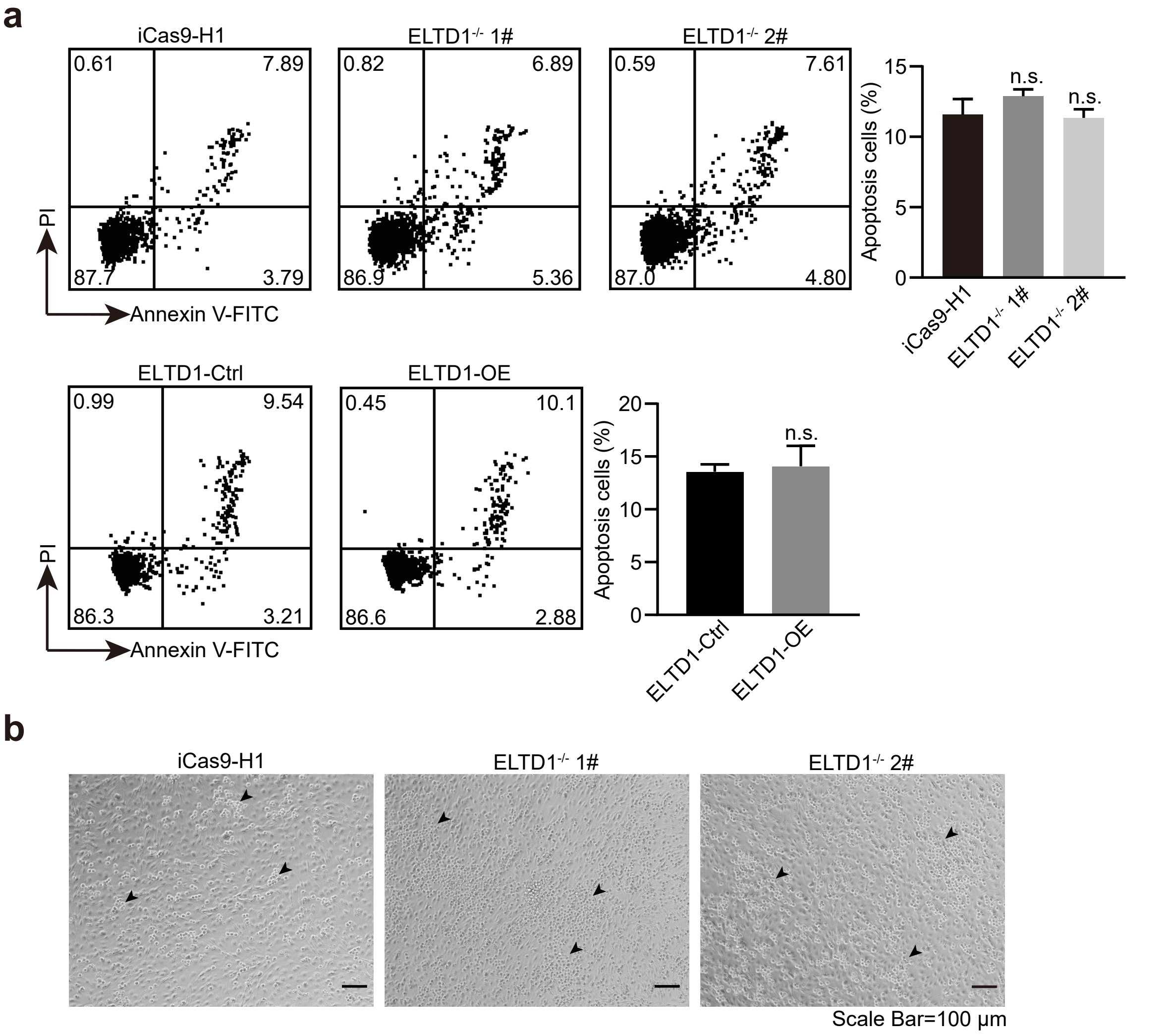

# Supplementary Figure 9

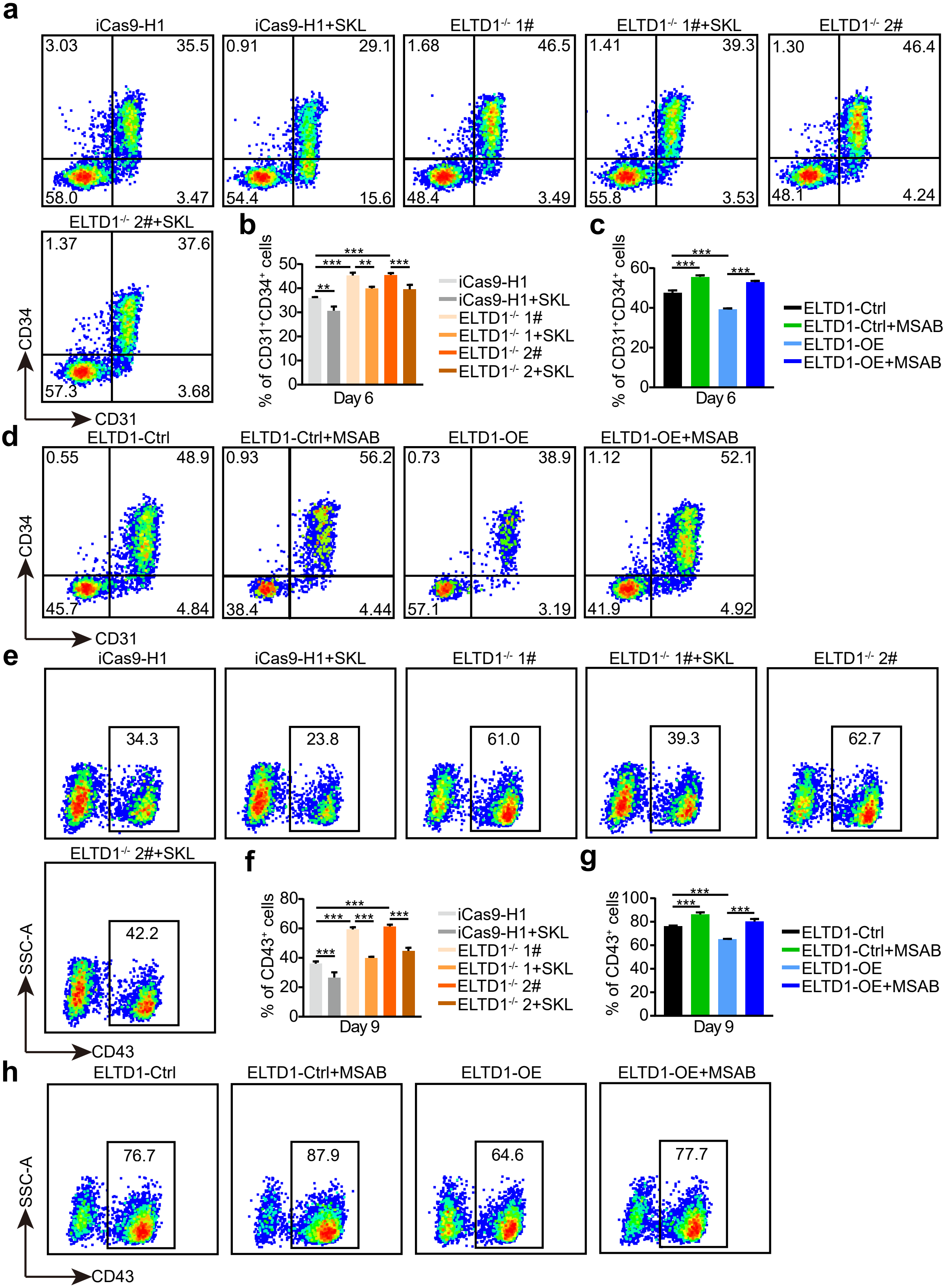

## SUPPLEMENTARY FIGURE LEGENDS

**Supplementary Fig. 1 The effectiveness of hematopoietic differentiation method in this study.**

a. qPCR was performed to analyze the mRNA levels of hematopoietic-related markers (APLNR, CD31, CD34, and CD43), axial mesoderm markers (SFRP2 and DCLK1), lateral plate mesoderm markers (CD309 and HAPLN1), paraxial mesoderm markers (MESP2 and MSGN1), intermediate mesoderm markers (CDH5 and OSR1), extra-embryonic mesoderm markers (IGF2 and LUM), and pluripotent markers (OCT4 and SOX2) during hESC hematopoietic differentiation. Results are shown as means ± SD; n=3. Statistical significance is indicated as compared to the preceding group: n.s., not significant, *$P < 0.05$, **$P < 0.01$, and ***$P < 0.001$.

b. Alkaline phosphatase activity of hESCs gradually declined during hESC hematopoietic differentiation. Scale bar: 100 μm.

**Supplementary Fig. 2 Mfuzz and GO analysis of DEGs in the process of hESC hematopoietic differentiation.**

a. The gene expression changes in cluster 1 identified by Mfuzz analysis.

b. GO analysis of biologic processes of the genes in cluster 1.

c. The gene expression changes in cluster 3 identified by Mfuzz analysis.

d. GO analysis of biologic processes of the genes in cluster 3.

e. The gene expression changes in cluster 4 identified by Mfuzz analysis.

f. GO analysis of biologic processes of the genes in cluster 4.

g. The gene expression changes in cluster 5 identified by Mfuzz analysis.

h. GO analysis of biologic processes of the genes in cluster 5.

i. The gene expression changes in cluster 6 identified by Mfuzz analysis.

j. GO analysis of biologic processes of the genes in cluster 6.

**Supplementary Fig. 3 The expression data of ELTD1 from The Human Protein Atlas database and the dynamic changes of ADGRL family members during hematopoietic differentiation.**

a. The expression information of ELTD1 in different cells of various tissues obtained from https://www.proteinatlas.org/. The image was adapted and modified from its original version, sourced from The Human Protein Atlas.

b. The expression level of ELTD1 in different cell types obtained from https://www.proteinatlas.org/. The image was adapted from The Human Protein Atlas and has been modified from its original form.

c. Heatmap showing expression changes of ADGRL family members during hESC hematopoietic differentiation.

**Supplementary Fig. 4 Construction of ELTD1 knockdown, overexpression and deletion hESCs.**

a. qPCR showing ELTD1 transcript knockdown efficiency in hESCs after infection with ELTD1 shRNA lentivirus. Results are shown as means ± SD; n=3. n.s., not significant, **P* < 0.05, ***P* < 0.01, and ****P* < 0.001, compared with that of the ELTD1-shCtrl group.

b. qPCR analysis showing ELTD1 transcript overexpression in hESCs. Results are shown as means ± SD; n=3. n.s., not significant, **P* < 0.05, ***P* < 0.01, and ****P* < 0.001, compared with that of the H1-ELTD1-Ctrl group.

c. Western blotting analysis of the expression of ELTD1 in H1-ELTD1-Ctrl and H1-ELTD1-OE.

d. qPCR showed the mRNA levels of OCT4, SOX2, and NANOG in H1-ELTD1-Ctrl and H1-ELTD1-OE. Results are shown as means ± SD; n=3. n.s., not significant, **P* < 0.05, ***P* < 0.01, and ****P* < 0.001, compared with that of the H1-ELTD1-Ctrl group.

e. Schematic representation of the CRISPR-Cas9 targeting strategy. The slash mark and scissors represent the correct Cas9-mediated cleavage position. The protospacer-adjacent motif (PAM) sequence is marked in red.

f. Sanger sequencing analysis confirming the mutation sites in ELTD1$^{-/-}$ 2# hESCs.

g. cDNA mutation of ELTD1$^{-/-}$ 2# was tested by qPCR. The results validate the cDNA mutation introduced in ELTD1$^{-/-}$ 2#. Results are shown as means ± SD; n=3. n.s., not significant, **P* < 0.05, ***P* < 0.01, and ****P* < 0.001, compared with that of the iCas9-H1 group.

h. Gel electrophoresis analysis confirming the mutated region in qPCR products from the cDNA

of ELTD1$^{-/-}$ 2# hESCs.

**Supplementary Fig. 5 ELTD1 deletion did not affect hESC morphology, pluripotency and differentiation capacity.**

a. ELTD1$^{-/-}$ 1# and ELTD1$^{-/-}$ 2# possesses the normal hESC morphology. Scale bar: 100 μm.

b. qPCR showed the mRNA levels of OCT4, SOX2, and NANOG in iCas9-H1, ELTD1$^{-/-}$ 1# and ELTD1$^{-/}$- 2# hESCs. Results are shown as means ± SD; n=3. n.s., not significant, *$P < 0.05$, **$P < 0.01$, and ***$P < 0.001$, compared with that of the iCas9-H1 group.

c. Western blotting detection of OCT4, SOX2, and NANOG in iCas9-H1, ELTD1$^{-/-}$ 1# and ELTD1$^{-/-}$ 2# hESCs.

d. Hematoxylin and eosin (H&E) staining of the teratomas derived from ELTD1$^{-/-}$ 1# and ELTD1$^{-/-}$ 2# hESCs. Arrows indicate representative tissues or cells from the three germ layers: endoderm-derived respiratory-type epithelia, mesoderm-derived cartilage tissue, and ectoderm-derived neuroepithelial cells. Scale bar: 100 μm.

e. The karyotype analysis revealed a normal chromosomal complement with no detectable abnormalities, confirming the genetic stability of the ELTD1$^{-/-}$ #2 cell line.

f. qPCR showed OCT4, SOX2, and NANOG expression changes during hESC hematopoietic differentiation. Results are shown as means ± SD; n=3. Statistical significance is indicated as compared to the preceding group: n.s., not significant, *$P < 0.05$, **$P < 0.01$, and ***$P < 0.001$.

**Supplementary Fig. 6 ELTD1 suppression enhances the specification of HEPs and subsequent hematopoietic differentiation from H9-hESCs.**

a. Flow cytometry analysis of mesoderm cells at day 3 of early hematopoietic differentiation. The percentage of APLNR$^{+}$ cells was quantified to evaluate the formation of mesoderm cells. Results are shown as means ± SD; n=3. n.s., not significant, *$P < 0.05$, **$P < 0.01$, and ***$P < 0.001$, compared with that of the H9-ELTD1-shCtrl group.

b. Flow cytometry analysis of HEPs at day 6 of early hematopoietic differentiation. The percentage of CD31$^{+}$CD34$^{+}$ cells was quantified to evaluate the formation of HEPs. Results are

shown as means ± SD; n=3. n.s., not significant, *$P < 0.05$, **$P < 0.01$, and ***$P < 0.001$, compared with that of the H9-ELTD1-shCtrl group.

c. Flow cytometry analysis of hematopoietic cells at day 9 of early hematopoietic differentiation. The percentage of $CD43^+$ cells was quantified to evaluate the formation of hematopoietic cells. Results are shown as means ± SD; n=3. n.s., not significant, *$P < 0.05$, **$P < 0.01$, and ***$P < 0.001$, compared with that of the H9-ELTD1-shCtrl group.

d. Flow cytometry analysis of mesoderm cells at day 3 of early hematopoietic differentiation. The percentage of $APLNR^+$ cells was quantified to evaluate the formation of mesoderm cells. Results are shown as means ± SD; n=3. n.s., not significant, *$P < 0.05$, **$P < 0.01$, and ***$P < 0.001$, compared with that of the H9-ELTD1-Ctrl group.

e. Flow cytometry analysis of HEPs at day 6 of early hematopoietic differentiation. The percentage of $CD31^+CD34^+$ cells was quantified to evaluate the formation of HEPs. Results are shown as means ± SD; n=3. n.s., not significant, *$P < 0.05$, **$P < 0.01$, and ***$P < 0.001$, compared with that of the H9-ELTD1-Ctrl group.

f. Flow cytometry analysis of $CD43^+$ hematopoietic cells at day 9 of early hematopoietic differentiation. The percentage of $CD43^+$ cells was quantified to evaluate the formation of HEPs. Results are shown as means ± SD; n=3. n.s., not significant, *$P < 0.05$, **$P < 0.01$, and ***$P < 0.001$, compared with that of the H9-ELTD1-Ctrl group.

**Supplementary Fig. 7 ELTD1 suppression enhances the specification of HEPs and subsequent hematopoietic differentiation from iPSCs.**

a. Flow cytometry analysis of mesoderm cells at day 3 of early hematopoietic differentiation. The percentage of $APLNR^+$ cells was quantified to evaluate the formation of mesoderm cells. Results are shown as means ± SD; n=3. n.s., not significant, *$P < 0.05$, **$P < 0.01$, and ***$P < 0.001$, compared with that of the iPSC-ELTD1-shCtrl group.

b. Flow cytometry analysis of HEPs at day 6 of early hematopoietic differentiation. The percentage of $CD31^+CD34^+$ cells was quantified to evaluate the formation of HEPs. Results are shown as means ± SD; n=3. n.s., not significant, *$P < 0.05$, **$P < 0.01$, and ***$P < 0.001$, compared with that of the iPSC-ELTD1-shCtrl group.

c. Flow cytometry analysis of hematopoietic cells at day 9 of early hematopoietic differentiation. The percentage of $CD43^+$ cells was quantified to evaluate the formation of hematopoietic cells. Results are shown as means ± SD; n=3. n.s., not significant, *$P < 0.05$, **$P < 0.01$, and ***$P < 0.001$, compared with that of the iPSC-ELTD1-shCtrl group.

d. Flow cytometry analysis of mesoderm cells at day 3 of early hematopoietic differentiation. The percentage of $APLNR^+$ cells was quantified to evaluate the formation of mesoderm cells. Results are shown as means ± SD; n=3. n.s., not significant, *$P < 0.05$, **$P < 0.01$, and ***$P < 0.001$, compared with that of the iPSC-Ctrl group.

e. Flow cytometry analysis of HEPs at day 6 of early hematopoietic differentiation. The percentage of $CD31^+CD34^+$ cells was quantified to evaluate the formation of HEPs. Results are shown as means ± SD; n=3. n.s., not significant, *$P < 0.05$, **$P < 0.01$, and ***$P < 0.001$, compared with that of the iPSC-Ctrl group.

f. Flow cytometry analysis of $CD43^+$ hematopoietic cells at day 9 of early hematopoietic differentiation. The percentage of $CD43^+$ cells was quantified to evaluate the formation of HEPs. Results are shown as means ± SD; n=3. n.s., not significant, *$P < 0.05$, **$P < 0.01$, and ***$P < 0.001$, compared with that of the iPSC-Ctrl group.

**Supplementary Fig. 8 Enhanced hematopoietic cell generation and unchanged apoptosis levels in HEPs derived from ELTD1-deleted hESCs.**

a. Knockout or overexpression of ELTD1 does not affect the apoptosis levels of $CD31^+CD34^+$ HEPs generated from hESCs. Results are shown as means ± SD; n=3. n.s., not significant, *$P < 0.05$, **$P < 0.01$, and ***$P < 0.001$, compared with that of the iCas9-H1 group.

b. Bright-field photomicrographs illustrate that $CD31^+CD34^+$ HEPs derived from the $ELTD1^{-/-}$ group exhibit a significantly increased capacity to generate hematopoietic cells compared to iCas9-H1 group. Scale bar: 100 μm.

**Supplementary Fig. 9 ELTD1 mediates HEP specification from hESCs via Wnt signaling.**

a. Flow cytometry detection of $CD31^+CD34^+$ HEPs at day 6 of early hematopoietic differentiation after SKL2001 treatment (5 μM) or not.

b. Statistical analysis of the flow cytometry data presented in Fig. S9a. Results are shown as means ± SD; n=3. n.s., not significant, *$P < 0.05$, **$P < 0.01$, and ***$P < 0.001$.

c. Statistical analysis of the flow cytometry data presented in Fig. S9d. Results are shown as means ± SD; n=3. n.s., not significant, *$P < 0.05$, **$P < 0.01$, and ***$P < 0.001$.

d. Flow cytometry detection of $CD31^+CD34^+$ HEPs at day 6 of early hematopoietic differentiation after MSAB treatment (1 μM) or not.

e. Flow cytometry detection of $CD43^+$ hematopoietic cells at day 9 of early hematopoietic differentiation after SKL2001 treatment (5 μM) or not.

f. Statistical analysis of the flow cytometry data presented in Fig. S9e. Results are shown as means ± SD; n=3. n.s., not significant, *$P < 0.05$, **$P < 0.01$, and ***$P < 0.001$.

g. Statistical analysis of the flow cytometry data presented in Fig. S9h. Results are shown as means ± SD; n=3. n.s., not significant, *$P < 0.05$, **$P < 0.01$, and ***$P < 0.001$.

h. Flow cytometry detection of $CD43^+$ hematopoietic cells at day 9 of early hematopoietic differentiation after MSAB treatment (1 μM) or not.

**Supplementary Table 1.** Sequences of primers, sgRNAs, siRNAs and shRNAs used in this study.

| *Gene* | *Species* | *Forward Primer (5'-3')* | *Reverse Primer (5'-3')* |
|---|---|---|---|
| ELTD1 | Human | TCAGATCCAGCAGTAACCAAGA | CTAAATGGCAGTTTGCATTCACA |
| APLNR | Human | CTCTGGACCGTGTTTCGGAG | GGTACGTGTAGGTAGCCCACA |
| CD309 | Human | GGCCCAATAATCAGAGTGGCA | CCAGTGTCATTTCCGATCACTTT |
| CD31 | Human | AACAGTGTTGACATGAAGAGCC | TGTAAAACAGCACGTCATCCTT |
| CD34 | Human | CTACAACACCTAGTACCCTTGGA | GGTGAACACTGTGCTGATTACA |
| CD43 | Human | GCTGGTGGTAAGCCCAGAC | GGCTCGCTAGTAGAGACCAAA |
| OCT4 | Human | TCTATTTGGGAAGGTATTCAGC | ATTGTTGTCAGCTTCCTCCA |
| NANOG | Human | AAGGTCCCGGTCAAGAAACAG | CTTCTGCGTCACACCATTGC |
| SOX2 | Human | CCCAGCAGACTTCACATGT | CCTCCCATTTCCCTCGTTTT |
| AXIN2 | Human | CAACACCAGGCGGAACGAA | GCCCAATAAGGAGTGTAAGGACT |
| CCND1 | Human | GCTGCGAAGTGGAAACCATC | CCTCCTTCTGCACACATTTGAA |
| BIRC5 | Human | AGGACCACCGCATCTCTACAT | AAGTCTGGCTCGTTCTCAGTG |
| TCF7L1 | Human | CAAGGTGGTGGCTGAGTG | TGGGTAGAGCTGCGAGTG |
| HPIP | Human | ATGGGTCTTCTGCTGGACAA | CAGGCTCTGAAGCTCTTCCTT |
| SFRP2 | Human | ACGGCATCGAATACCAGAACA | CTCGTCTAGGTCATCGAGGCA |
| HAPLN1 | Human | TCTGGTGCTGATTTCAATCTGC | TGCTTGGATGTGAATAGCTCTG |
| OSR1 | Human | CGGTGCCTATCCACCCTTC | GCAACGCGCTGAAACCATA |
| CDH5 | Human | TTGGAACCAGATGCACATTGAT | TCTTGCGACTCACGCTTGAC |
| LUM | Human | TAACTGCCCTGAAAGCTACCC | GGAGGCACCATTGGTACACTT |
| IGF2 | Human | GTGGCATCGTTGAGGAGTG | CACGTCCCTCTCGGACTTG |
| MESP2 | Human | CACGACCACTGGATCTTCGC | AACCCGACGAATCGGAGGA |
| MSGN1 | Human | AACCTGCGCGAGACTTTCC | GTCTGTGAGTTCCCCGATGTA |
| ACTIN | Human | CATGTACGTTGCTATCCAGGC | CTCCTTAATGTCACGCACGAT |

| *Gene* | *Target* | *Forward Primer (5'-3')* | *Reverse Primer (5'-3')* |
|---|---|---|---|
| ELTD1 | WT-mutation region | CTAAGTGCTCTCTTGGGAAA | AGTTAGTTTCTGGAGGACAG |
| ELTD1 | KO1-mutation region | CAATGGAATTGAAGCCTGCT | TGTCTTGGTTACTGCTGGAT |
| ELTD1 | KO2-mutation region | CCTGCTATTGCAACATGG | GTCCTTGGCTGAGATAGTG |

| *Gene* | *sgRNA* | *(5'-3')* |
|---|---|---|
| ELTD1 | sgRNA-1 | GAAGCCTGCTATTGCAACAT |
| ELTD1 | sgRNA-2 | GCAAAATGTGAAATACGCAA |

| *Gene* | *siRNA* | *Forward Primer (5'-3')* | *Reverse Primer (5'-3')* |
|---|---|---|---|
| ELTD1 | siRNA-1 | UGGUCCUUCCAUUGGUAUUAA | UUAAUACCAAUGGAAGGACCA |

| | | | |
|---|---|---|---|
| ELTD1 | siRNA-2 | CACACCUCAUGCCGCUGUAAU | AUUACAGCGGCAUGAGGUGUG |

| ***Gene*** | ***shRNA*** | ***(5'-3')*** |
|---|---|---|
| ELTD1 | shctrl | CCGGGGTTCTCCGAACGTGTCACGTCTCGAGACGTGACACGTTCGGAGAACCTTTTTG |
| ELTD1 | sh1 | CCGGTGGTCCTTCCATTGGTATTAACTCGAGTTAATACCAATGGAAGGACCATTTTTT |
| ELTD1 | sh2 | CCGGCACACCTCATGCCGCTGTAATCTCGAGATTACAGCGGCATGAGGTGTGTTTTTT |
| ELTD1 | sh3 | CCGGGCACTAGGATACAGATATTATCTCGAGATAATATCTGTATCCTAGTGCTTTTTT |
| HPIP | sh1 | CCGGGGCTGAGCACTGGAAACATAACTCGAGTTATGTTTCCAGTGCTCAGCCTTTTTT |
| HPIP | sh2 | CCGGAGGCATTAAGGCAAGAGTTAGCTCGAGCTAACTCTTGCCTTAATGCCTTTTTTT |

**Supplementary Table 2.** Resources of key reagents used in this study.

| REAGENT/ RESOURCES | SOURCE | CATALOGUE |
|---|---|---|
| ***Antibodies*** | | |
| OCT4 (EPR17929) | Abcam | ab181557, RRID: AB_2687916 |
| SOX2 (SP76) | Abcam | ab93689, RRID: AB_10562630 |
| NANOG (EPR2027(2)) | Abcam | ab109250, RRID: AB_10863442 |
| Phospho-β-catenin (Ser33/37/Thr41) | Cell Signaling Technology | 9561 |
| Phospho-β-catenin (Ser45) | Cell Signaling Technology | 9564 |
| β-Actin (13E5) | Cell Signaling Technology | 4970, RRID: AB_2223172 |
| β-Actin (High Dilution) | ABclonal | AC026, RRID: AB_2768234 |
| HPIP | Proteintech | 12102-1-AP, RRID: AB_2160484 |
| ELTD1 (clone CL4164) | Atlas | AMAb91268, RRID: AB_2665875 |
| ELTD1 | Abmart | PH2541 |
| CD309/FLK1/KDR/VEGFR2 (D-8) | Santa Cruz Biotechnology | sc393163, RRID: AB_2920761 |
| CD31 (JC/70A) | Abcam | ab9498, RRID: AB_307284 |
| CD34 (EP373Y) | Abcam | ab81289, RRID: AB_1640331 |
| CD43 (EPR21904) | Abcam | ab235453 |
| Alexa Fluor 488 Goat anti-Rabbit | Thermo Fisher Scientific | A11008, RRID: AB_143165 |
| Alexa Fluor 647 Goat anti-Mouse | Thermo Fisher Scientific | A21236, RRID: AB_2535805 |
| APC anti-APLNR (clone 72133R) | R&D systems | FAB8561A, RRID: AB_3653439 |
| PerCP/Cyanine5.5 anti-human CD309 (clone 7D4-6) | Biolegend | 359908, RRID: AB_2563487 |
| FITC anti-human CD31 (clone WM59) | Biolegend | 303103, RRID: AB_314329 |
| BV421 anti-human CD31 (clone WM59) | Biolegend | 303123, RRID: AB_2562179 |
| APC anti-human CD34 (clone 561) | Biolegend | 343608, RRID: AB_2228972 |
| APC anti-human CD43 (clone CD43-10G7) | Biolegend | 343206, RRID: AB_2194072 |

| | | |
|---|---|---|
| ***Chemicals, Media and Kits*** | | |
| CHIR-99021 | TargetMol | T2310 |
| SKL-2001 | MCE | HY-101085 |
| IWR-1 | MCE | HY-12238 |
| MSAB | MCE | HY-120697 |
| G-418 disulfate | MCE | HY-17561 |
| Puromycin dihydrochloride | TargetMol | T2219 |
| Blasticidin S hydrochloride | MCE | HY-103401 |
| Doxycycline | TargetMol | T1687 |
| Anti-FLAG M2 Magnetic Beads | Millipore | M8823 |
| Matrigel | Corning | 354277 |
| mTeSR™1 Complete Kit | Stem Cell | 85850 |
| STEMdiff™ Hematopoietic Kit | Stem Cell | 05310 |
| ReLeSR | Stem Cell | 100-0483 |
| Accutase | Stem Cell | 07920 |
| CloneR | Stem Cell | 05889 |
| MethoCult™ | Stem Cell | H4435 |
| DMEM | Gibco | 11965092 |
| DMEM/F-12 | Gibco | 11320033 |
| FBS | Gibco | A5256701 |
| RNAiso Plus | Takara | 9109 |
| RIPA Lysis Buffer | Absin | abs9230 |
| Protease and phosphatase inhibitor cocktail | Beyotime | P1045 |
| Paraformaldehyde, 4% | Solarbio | P1110 |
| DAPI Fluoromount-G | Yeasen | 36308ES11 |
| ECL Enhanced Plus Kit | ABclonal | RM00021P |
| HiScript III RT SuperMix for qPCR | Vazyme | R323-01 |
| ChamQ Universal SYBR qPCR Master Mix | Vazyme | Q711-02 |
| PureLink HiPure Plasmid Maxiprep Kit | Thermo Fisher Scientific | K210007 |
| FITC Annexin V Apoptosis Detection Kit I | BD Pharmingen | 556547 |
| ***Plasmids, sgRNAs, siRNAs, and lentivirus*** | | |
| ELTD1-sgRNAs | Youkang Biotech Co., Ltd. | N/A |
| ELTD1-shRNA-plasmids | Transheep Biotech Co., Ltd. | N/A |
| ELTD1-OE-plasmids | Transheep Biotech Co., Ltd. | N/A |
| ELTD1-siRNAs | Transheep Biotech Co., Ltd. | N/A |
| HPIP-shRNA-lentivirus | Transheep Biotech Co., Ltd. | N/A |
| LEF1-OE-lentivirus | Transheep Biotech Co., Ltd. | N/A |
